# Epidemiology of human and animal brucellosis in Kenya: A One Health meta-regression and network analysis

**DOI:** 10.1016/j.onehlt.2026.101390

**Published:** 2026-03-18

**Authors:** Martin Wainaina, Joseph Samuel Kimatu, Benson Rukwaro, Elizabeth Anne Jessie Cook

**Affiliations:** a Department of Biological Safety, German Federal Institute for Risk Assessment, Berlin, Germany; b International Livestock Research Institute, Nairobi, Kenya

**Keywords:** Brucellosis, One Health, Network analysis, Surveillance, Meta-analysis, Kenya

## Abstract

**Background:**

Brucellosis is a prioritised zoonotic disease in Kenya, but an in-depth quantitative review of its presence in humans and animals is currently lacking.

**Methods:**

Systematic literature searches were performed in African Journals Online (AJOL), Embase, PubMed, Scopus, and Web of Science databases. Relevant data were extracted from prevalence studies and random-effects meta-analyses were applied to estimate pooled prevalence by sample type and diagnostic methods for five key species. Between-study heterogeneity was explored using meta-regression models. A seroprevalence-weighted bipartite network analysis was used to link brucellosis research on various host species to livelihood zones in Kenya.

**Results:**

We found 65 brucellosis prevalence studies that increased with time, spanning 1969–2024 (55 years) and consisting of samples from 20,944 humans, 57,340 domestic animals, 727 wildlife from 16 species, and 235 Hippoboscid flies (*Hippobosca* spp.). Sampling methodologies comprised non-probability (*n* = 29, 44.6%), mixed (i.e., both probability and non-probability, *n* = 17, 26.2%), probability (*n* = 16, 24.6%), and census approaches (*n* = 3, 4.6%), and sample size calculations were reported in 38.5% (*n* = 25) of studies. The overall seroprevalence using indirect serological tests on blood samples was: 4.8% (95% CI: 3.1–7.3) in sheep, 7.9% (95% CI: 4.5–13.6) in goats, 10.2% (95% CI: 7.0–14.8) in cattle, 10.8% (95% CI: 4.4–24.2) in humans from community settings, and 13.2% (95% CI: 7.6–21.9) in camels. There was high between-study heterogeneity in all hosts (*I*^2^ ≥ 75.0). The test category was a source of between-study heterogeneity, with direct tests having 3.37 times higher odds (95% CI: 1.66–6.85, *p*-value = 0.0009) of determining positive results in animals than indirect tests. There was no clear difference between seroprevalence in domestic and wild animals (*p*-value = 0.73). Network analysis revealed that pastoral (strength = 130.0) and agropastoral zones (strength = 69.6) had the highest brucellosis presence, with humans (strength = 50.2) and cattle (strength = 38.2) showing the highest cumulative seroprevalence among hosts. Goats were the most central hosts (betweenness = 31), and all wildlife were peripheral nodes (betweenness = 0) despite considerable seroprevalence. Over half (55.4%) of possible host-zone combinations were unstudied

**Conclusion:**

There is widespread brucellosis, especially in pastoral and agropastoral systems. Current published evidence has considerable geographic gaps and under-represents wildlife. The use of probability sampling approaches in future epidemiological studies, where possible, could ensure results are more representative of the population. A One Health approach integrating human, livestock, and wildlife populations is required in future studies for comprehensive risk assessment of brucellosis in Kenya. Lastly, continued resource allocation towards brucellosis prevention and control is required to lower disease presence.

## Highlights

• First quantitative One Health review of brucellosis in Kenya.
• Pooled seroprevalence ranged from 4.8 to 13.2% in humans and four domestic species.
• Network analysis identified pastoral/agropastoral zones and goats as key nodes.
• Diagnostic test type was an important source of between-study heterogeneity.
• Several geographical zones and wildlife are under-researched.

## 1 Introduction

Brucellosis is an important zoonosis with global distribution. It is caused by pathogenic bacteria of the genus *Brucella*. The genus consists of classical/core species (*B. melitensis*, *B. abortus*, *B. suis*, *B. canis*, *B. ovis*, and *B. neotomae*), marine (*B. pinnipedialis* and *B. ceti*), and novel species (*B. inopinata*, *B. papionis*, *B. vulpis*, and *B. microti*), with some strains awaiting taxonomic assignment [1]. Several species have zoonotic potential, but *B. melitensis*, *B. abortus* and *B. suis* are responsible for most cases of human brucellosis globally. Brucellae exhibit a characteristic host preference, with *B. abortus* being commonly associated with cattle, *B. melitensis* with small ruminants, and *B. suis* with pigs, hare, and caribou [2].

The clinical presentation of human brucellosis can vary from mild flu-like illness with accompanying symptoms such as fever, abdominal pain, sweats, myalgia, arthralgia, and general malaise [3]. The infection can be mild and self-limiting in some patients, but untreated brucellosis can progress to chronic and severe forms with multi-system involvement, leading to debilitating illness and mortality [3]. Animals acquire brucellosis through contact with infected animals or wildlife reservoirs, ingestion of contaminated feed, vertically, and through venereal transmission [4], [5]. The disease can cause considerable economic losses from reproductive wastage and productivity losses in livestock [4]. Transmission to persons typically occurs through ingesting contaminated foods (e.g., raw milk and unpasteurised dairy products), or through contact with infected animals or their bodily fluids in occupationally-exposed slaughterhouse workers, veterinarians, farmers, hunters, etc. [6]. Brucellosis diagnosis can be done using direct detection methods which identify the pathogen in clinical samples during infection, or indirectly where antibody responses to the pathogen are identified [7].

Brucellosis is an important cause of non-malarial fevers in Africa [8], and its lack of distinguishing clinical features makes differential diagnosis from other febrile illnesses like leptospirosis difficult [9]. In Kenya, brucellosis is a zoonosis that was prioritised for targeted control and management based on the observed public health burden and socio-economic impact on animal and human populations [10]. The inaugural systematic brucellosis review in Kenya was an impactful work that revealed the widespread occurrence in humans and animals [11]. Nearly a decade later, several studies have been performed filling the identified research gaps. A quantitative synthesis of Kenya's brucellosis situation is however still lacking and long overdue. We address this knowledge gap in this study by (i). performing a meta-analysis to generate pooled prevalence estimates in important hosts, (ii). conducting meta-regression analyses to identify sources of between-study heterogeneity, and (iii). exploring the interconnectedness of brucellosis research to determine the most consequential hosts and transmission zones.

## 2 Methods

### 2.1 Literature searches

We searched for literature using the Preferred Reporting Items for Systematic reviews and Meta-Analyses (PRISMA) guidelines [12], and used a pre-registered protocol [13]. The search databases were African Journals Online (AJOL), Embase, PubMed, Scopus, and Web of Science. We used Boolean operators on the search strings to combine terms related to the pathogen and disease (brucella, brucellosis, and synonyms), epidemiological measures (prevalence, incidence, risk factors, etc.), Kenya, and host species (humans, livestock, wildlife) (Supplementary Information). The searches were conducted without language or time restrictions.

### 2.2 Study selection

Studies were imported into Rayyan web platform (https://www.rayyan.ai/), where they were deduplicated and screened by title and abstract for inclusion with two authors (J.S.K. and B.R.). Any disagreements on the eligibility were settled through consensus and in consultation with a third author (M.W.). Screening using full texts was performed on potentially eligible studies, and the final list comprising studies that met the inclusion criteria was exported. The reference sections of relevant reviews were manually screened to identify additional eligible studies. Translation was performed using DeepL translation tool (https://www.deepl.com/).

### 2.3 Eligibility criteria

Studies were eligible if they reported the positives and totals or prevalence (with uncertainty range) of brucellosis in Kenyan humans, animals or foods using diagnostic tests (serological, culture, or molecular methods). Eligible study designs included cross-sectional studies and baseline populations of longitudinal studies. Both peer-reviewed publications and abstracts were included if methodological details were sufficient, but grey literature (theses) were rejected. All human populations (general, occupational, clinical) and animal species (livestock, wildlife, vectors) within Kenya were eligible. Lastly, studies using probabilistic or non-probabilistic sampling approaches were included.

Studies were excluded if they: (i). were conducted entirely outside Kenya, lacked Kenya-specific data, or investigated non-Kenyan populations, e.g., travellers; (ii). were not natural infections; (iii). reported duplicate data from previously published publications (the most comprehensive report was retained); (iv). did not report prevalence or insufficient data for its calculation; (v). used non-representative samples (e.g., case reports, case series, outbreak investigations); (vi). did not utilise laboratory or field diagnostics; and (vii). contained no original data (reviews, commentaries, editorials, etc.).

### 2.4 Meta-analysis

Prevalence data were extracted as raw counts (positive/total) where available, or as prevalence estimates and 95% confidence intervals (CI) for design-adjusted studies without raw counts. For the latter, standard errors (SE) were calculated from the confidence intervals using the formula $\mathrm{SE}=\left(\text{Upper}\;\mathrm{CI}-\text{Lower}\;\mathrm{CI}\right)/\left(2\times 1.96\right)$, and values were logit-transformed before combining with the other studies. When multiple diagnostic tests were reported with no overall prevalence, results from more common tests (e.g., the Rose Bengal test, IgG tests) were preferred to harmonise the data, and those of the Febrile *Brucella* antigen test (FBAT) were avoided because of its limited diagnostic performance.

Effect sizes and variances were estimated using the *escalc* function of the *metafor* package in R statistical environment. A default continuity correction of 0.5 was used to stabilise estimates for studies with zero prevalence. Random-effects models were fitted to pool study prevalence estimates using the restricted maximum-likelihood (REML) estimator using the *meta* package. Between-study heterogeneity was quantified using Higgins' *I*^2^ statistic and Tau squared (τ^2^). A Hartung-Knapp adjustment was applied to the models to estimate more reliable confidence intervals given the low study numbers. This was done separately for the various human and animal species with study numbers (k) we deemed sufficient (k ≥ 10). Subgroup analyses were conducted according to diagnostic test and sample types. Separate analyses were performed for studies with probability sampling methods to obtain more representative summary estimates.

### 2.5 Meta-regression analysis

We fitted random-effects meta-regression models using the REML estimator and a Hartung-Knapp adjustment to explore sources of between-study heterogeneity using the *metafor* package. Separate models were fitted for cattle, humans, goats, sheep and camels. A final combined animal model was fitted with domestic and wildlife categories comprising the “host type” variable. Variables were chosen *a priori* based on biological plausibility and data availability, and these included: test category (direct, indirect, and both detection methods), sample type (blood, milk [from individual animals], pooled milk [at household and market level as these have unknown numbers of constituent animals], cervical swabs, and semen), host type, and population type (febrile versus “others” in human studies only). The study year was included as a continuous variable using the year data collection ended and was centred at 2000 to improve interpretability.

Model building began with a full model that included all pre-specified variables (“population type”, “study end date (centred)” and “test category” for humans; and “sample type”, “study end date (centred)” and “test category” in animal models. The “host type” variable was applied in the combined animal model, and interaction between “sample type” and “test category” was also included due to the higher study numbers (k) that reduce chances of overfitting. Models were simplified by removing the least informative covariate, while checking for confounding effects. Akaike's Information Criterion (AIC), residual heterogeneity (τ^2^), and the proportion of explained heterogeneity (R^2^) were used to compare and determine the final models. The moderator coefficients were exponentiated to obtain odds ratios and their 95% confidence intervals. Residual heterogeneity (τ^2^, *I*^2^, R^2^) and the overall *p*-values of variables were reported for each final model. Model diagnostics were performed using the leave-one-out influence analysis using the *influence* function. Publication bias analyses were avoided *a posteriori* due to the high heterogeneity observed.

### 2.6 Network analysis

A bipartite network linking the hosts and livelihood zones was fitted. Only studies reporting serological results disaggregated by location and host species with available positives and totals or prevalence and confidence intervals were utilised. To ensure more representative and homogeneous data, we excluded studies on milk samples and those utilising direct detection methods from these analyses.

Locations were categorised into 5 livelihood zones [Agropastoral ASAL; Coastal Marginal/Medium Mixed; High/Medium Potential Mixed (Highlands/West); Lakeshore/Fishing; and Pastoral ASAL] (Supplementary Information). The seroprevalence was pooled using random-effects meta-analysis for each livelihood zone-host combination using the methods previously outlined. Combinations with only one study used the observed prevalence without meta-analytic pooling, and those with no studies were classified as research gaps. The bipartite network was constructed using *igraph* [14] and visualised using *ggraph* packages [15]. Nodes represented livelihood zones and host species, and edges (i.e., connections between nodes) were weighted by the pooled seroprevalence. Network- and node-level metrics were computed to determine the research landscape and priorities (Table 1) [16].

**Table 1:** Network metrics used for assessing the relationship between the hosts and locations (categorised into 5 livelihood zones), with edges weighted by the pooled seroprevalence. Nodes are all the entities in a network (divided into hosts and locations in this study) and edges are the connections between two nodes.

Metric	Description	Interpretation
Degree	Number of edges connected to a node.	**Livelihood zones:** Number of host species studied in a zone (the breadth of research). **Host species:** Number of zones where a species has been studied (geographic coverage).
Strength	Sum of edge weights connected to a node. These were weighted using the pooled prevalence.	Sum of the pooled seroprevalences (%) for all connections of a node. If higher, the node is surrounded by edges with more infection overall. This can therefore be an index to the presence of brucellosis in a node.
Mean edge weight	The mean edge weight obtained by dividing the strength with the number of connections for a node	Average pooled prevalence (%) for the node's edges.
Betweenness	Number of shortest paths between all pairs of nodes that go through a given node.	**Livelihood zones:** if high, a zone acts as a bridge that connects otherwise separated animals. **Host species**: if high, the species is a key linkage for different zones.
Density	The number of observed edges (connections) divided by the maximum possible edges in a network.	This is a network-level metric that demonstrates how “full” or densely connected a network is.
Diameter	The longest of the shortest paths between any two nodes in a network.	A network-level metric that shows how many steps the most distant nodes are away from one another in the network.
Average path length	The mean number of steps along the shortest paths for all possible pairs of nodes.	A network-level metric that shows on average, how many steps it takes to get from one node to another through the network.

## 3 Results

### 3.1 Study characteristics

**Fig. 1:**
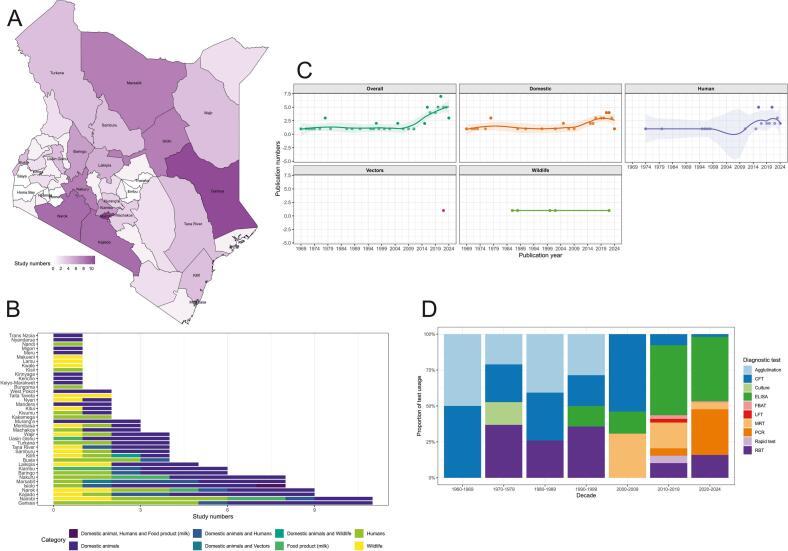
Brucellosis prevalence studies in Kenya (1969–2024). Spatial distribution **A.** by county, with seven counties having no studies (Bomet, Embu, Homa Bay, Nyamira, Siaya, Tharaka, and Vihiga), and **B.** One health study categories by county. **C.** Temporal distribution of publication numbers by host category, with smoothed curves (LOESS regression) and confidence intervals. **D.** The changes in diagnostic test usage by decade. Agglutination: Micro-, Serum and Tube agglutination tests, CFT: Complement fixation test, ELISA: Enzyme-linked immunosorbent assay, LFT: Lateral flow test, FBAT: Febrile *Brucella* antigen test, MRT: Milk ring test, PCR: Polymerase chain reaction, Rapid test: Rapid immunochromatographic flow assay, RBT: Rose Bengal test.

The primary search yielded 3184 results, but only 65 articles were finally included (Supplementary Information), encompassing 20,944 humans, 57,340 domestic animals from 6 species, 727 wildlife from 16 species and 235 hippoboscid fly samples. The publications were mostly journal articles (*n* = 64), spanned 1969–2024 (55 years), and were in English (n = 64) and German (*n* = 1). There was an overall increase in publication numbers with time, and in all host categories except wildlife and vectors (Fig. 1). Most publications also focussed on pastoral and agropastoral counties, and western and coastal regions received less attention (Fig. 1). Articles investigating domestic animals were the majority (*n* = 25) (Fig. 1). Cross-sectional study designs were the most frequent (*n* = 62), and the baseline prevalence of longitudinal studies (*n* = 3) were fewer (Supplementary Information). Non-probability sampling was most common (*n* = 29, 44.6%), followed by mixed approaches (i.e., both probability and non-probability, *n* = 17, 26.2%), probability sampling (*n* = 16, 24.6%), and census (n = 3, 4.6%). Only 25/65 studies (38.5%) reported conducting formal sample size calculations. Serological tests were the most widely used diagnostics, and the number of tests applied increased with time, with molecular approaches such as polymerase chain reaction (PCR) test being more popular in the last two decades (Fig. 1).

### 3.2 Summary estimates

The overall pooled prevalence varied with the species, and included sheep (5.7%, 95% CI: 3.5–9.1), goats (9.0%, 95% CI: 4.8–16.2), cattle (9.9%, 95% CI: 6.8–14.1), humans (10.9%, 95% CI: 6.6–17.5) and camels (14.5%, 95% CI: 8.2–24.3) in increasing order (Table 2). The overall summary estimates from studies using probability sampling methods only were: 5.2% (95% CI: 2.2–11.8) in sheep, 11.4% (95% CI: 4.5–26.0) in goats, 7.4% (95% CI: 3.7–14.5) in cattle, 10.4% (95% CI: 3.5–27.4) in humans, and 9.7% (95% CI: 1.2–49.9) in camels (Table 2). Overall estimates from studies using non-probability sampling methods only were: sheep (6.9%, 95% CI: 2.4–17.9), goats (11.4%, 95% CI: 2.2–42.1), cattle (8.8%, 95% CI: 4.6–16.2), humans (11.0%, 95% CI: 5.9–19.7) and camels (16.9%, 95% CI: 7.8–32.7) (Table 2). In general, direct detection methods showed higher pooled estimates than indirect methods. In humans, the highest prevalence in all studies was found in the community subgroup using direct detection methods (26.9%, 95% CI: 1.4–90.7), albeit with very wide confidence intervals and small study numbers (k = 3). There were statistically significant differences in pooled prevalence estimates from all studies when compared between the subgroups for cattle (*p*-value = 0.0056), goats (*p*-value = 0.0007), sheep (*p*-value = 0.03), and camels (*p*-value < 0.0001), but not in humans (*p*-value = 0.53), based on *χ*^2^ tests for subgroup differences (Q-tests for moderators) from the random-effects meta-analytic models (Supplementary Information). Lastly, the between-study heterogeneity observed was high in all species overall estimates, with the *I*^2^ values ranging from 89.2 to 98.4% (Table 2, Supplementary Information).

**Table 2:** Summary estimates of brucellosis prevalence from a random-effects meta-analysis with sample type (or population in humans) and diagnostic classification as subgroups.

			All studies †				Studies with probability sampling				Studies with non-probability sampling			
Host species	Sample/Population type	Diagnostic method	Studies (k)	Pooled prevalence % (95% CI) ⁎	τ^2^	*I*^2^ (%)	Studies (k)	Pooled prevalence % (95% CI) ⁎	τ^2^	*I*^2^ (%)	Studies (k)	Pooled prevalence % (95% CI) ⁎	τ^2^	*I*^2^ (%)
Cattle	Overall		39	9.9 (6.8–14.1)	1.19	97.8	11	7.4 (3.7–14.5)	0.88	96.7	12	8.8 (4.6–16.2)	1.05	94.0
	Blood	Direct	1	18.9 (14.3–24.5)										
	Milk	Direct	1	1.0 (0.1–13.8)										
	Pooled/bulk milk	Direct	1	1.2 (0.1–16.4)										
	Blood	Indirect	24	10.2 (7.0–14.8)	0.78	98.4	9	7.1 (2.7–17.1)	1.26	97.4	9	8.2 (4.9–13.5)	0.39	87.3
	Milk	Indirect	4	17.0 (6.9–36.2)	0.35	86.5	1	7.7 (4.8–12.2)			1	16.0 (12.4–20.3)		
	Pooled/bulk milk	Indirect	8	6.8 (1.4–27.4)	3.48	95.6	1	7.9 (4.2–14.5)			2	7.1 (0.0–100.0)	8.51	98.2
Humans	Overall		37	10.9 (6.6–17.5)	2.38	98.4	11	10.4 (3.5–27.4)	2.95	99.0	16	11.0 (5.9–19.7)	1.31	95.3
	Community	Direct	3	26.9 (1.4–90.7)	1.55	91.1	1	7.5 (3.4–15.7)			1	50.0 (24.4–75.6)		
	Febrile patients	Direct	8	10.9 (2.6–36.4)	2.61	94.8	1	15.5 (12.3–19.5)			5	20.2 (11.1–34.2)	0.27	92.4
	Febrile patients	Direct and Indirect	1	13.7 (11.8–15.9)			1	13.7 (11.8–15.9)						
	Community	Indirect	15	10.8 (4.4–24.2)	2.78	98.7	8	9.8 (1.9–38.3)	4.22	99.2	4	7.7 (0.7–50.7)	1.71	92.6
	Febrile patients	Indirect	10	7.8 (2.6–21.3)	2.51	99.0					6	5.9 (1.8–17.9)	1.28	92.1
Goats	Overall		23	9.0 (4.8–16.2)	1.62	95.7	5	11.4 (4.5–26.0)	0.62	98.3	9	11.4 (2.2–42.1)	3.56	89.7
	Blood	Direct	2	4.0 (0.0–100.0)	15.34	93.8					1	0.2 (0.0–3.2)		
	Cervical swabs	Direct	1	50.0 (24.4–75.6)							1	50.0 (24.4–75.6)		
	Milk	Direct	2	30.1 (0.0–100.0)	15.16	92.8					1	90.0 (32.6–99.4)		
	Pooled/bulk milk	Direct	1	5.2 (2.2–11.8)										
	Blood	Indirect	15	7.9 (4.5–13.6)	0.92	96.3	5	11.4 (4.5–26.0)	0.62	98.3	4	6.9 (1.7–24.3)	0.75	91.9
	Milk	Indirect	1	5.0 (2.3–10.7)							1	5.0 (2.3–10.7)		
	Pooled/bulk milk	Indirect	1	50.0 (5.9–94.1)							1	50.0 (5.9–94.1)		
Sheep	Overall		20	5.7 (3.5–9.1)	0.89	91.5	5	5.2 (2.2–11.8)	0.41	85.0	5	6.9 (2.4–17.9)	0.11	33.3
	Blood	Direct	2	6.1 (0.0–100.0)	10.93	91.5					1	0.5 (0.0–6.9)		
	Milk	Direct	1	6.3 (0.4–53.9)										
	Pooled/bulk milk	Direct	1	25.0 (1.3–89.1)										
	Semen	Direct	1	10.9 (8.1–14.7)										
	Blood	Indirect	14	4.8 (3.1–7.3)	0.36	75.0	5	5.2 (2.2–11.8)	0.41	85.0	3	8.1 (2.9–20.5)	0.03	0.0
	Milk	Indirect	1	3.6 (0.2–38.4)							1	3.6 (0.2–38.4)		
Camels	Overall		17	14.5 (8.2–24.3)	1.28	89.2	4	9.7 (1.2–49.9)	1.59	87.7	7	16.9 (7.8–32.7)	0.72	81.0
	Blood	Direct	2	60.5 (0.8–99.6)	0.22	67.0					1	50.0 (33.3–66.7)		
	Milk	Direct	1	12.5 (0.7–73.4)										
	Pooled/bulk milk	Direct	1	4.2 (0.3–42.5)										
	Blood	Indirect	10	13.2 (7.6–21.9)	0.45	81.3	4	9.7 (1.2–49.9)	1.59	87.7	4	14.0 (5.9–29.7)	0.28	74.5
	Milk	Indirect	1	8.3 (0.5–62.2)							1	8.3 (0.5–62.2)		
	Pooled/bulk milk	Indirect	2	4.5 (0.1–82.3)	0.00	0.0					1	9.1 (1.3–43.9)		

CI: confidence intervals.

⁎ Some estimates have exceedingly wide confidence intervals due to small study numbers.

† Comprises studies that used probability, non-probability, mixed sampling (i.e., probability and non-probability) and census approaches.

### 3.3 Meta-regression estimates

The meta-regression analysis revealed the diagnostic test variable was a significant source of between-study heterogeneity in camel studies, with direct tests having 7.27 times higher odds (95% CI: 2.16–24.54, *p*-value =0.0037) of determining positives than indirect ones. The final model for the camel data accounted for a large proportion of the heterogeneity (R^2^ = 70%) (Table 3). Influence analyses identified one potentially influential study, and its removal resulted in a change in the model metrics (τ^2^ = 0.16, R^2^ = 83.2%, odds for direct tests: 6.91, 95% CI [3.02 - 15.82], *p*-value = 0.0003).

**Table 3:** A summary of the multivariable meta-regression models investigating potential sources of the between-study heterogeneity in human and animal brucellosis in Kenya.

Host	Study number (k)†	Variable	Category	Coefficient (95% CI)	SE	*p*-value	Odds ratio (95% CI)	Final model (τ^2^, *I*^2^, R^2^)
Cattle	27		Intercept	−5.04 (−8.00 to – ‐2.08)	1.43	0.002		τ^2^ = 1.22, *I*^2^ = 98.1%, R^2^ **=** 10.8%
		Test category	Direct	Ref.				
			Indirect	2.65 (−0.24–5.55)	1.40	0.07	14.19 (0.78–256.90)	
		Study year‡	Centred at year 2000	0.03 (−0.02–0.08)	0.02	0.21	1.03 (0.98–1.08)	
Humans	30	Intercept		−1.89 (−3.07 to – ‐0.70)	0.58	0.003		τ^2^ = 2.62, *I*^2^ = 99.4%, R^2^ = 0.0%
		Population type	Other ⁎	Ref.				
			Febrile patients	−0.53 (−1.87–0.80)	0.65	0.42	0.59 (0.15–2.24)	
		Study year‡	Centred at year 2000	0.02 (−0.04–0.08)	0.03	0.60	1.02 (0.96–1.08)	
Goats	23	Intercept		−2.50 (−4.60 to – ‐0.40)	1.00	0.022		τ^2^ = 1.86; *I*^2^ = 98.7%, R^2^ **=** 0.0%
		Test category	Direct	Ref.				
			Indirect	−0.01 (−2.15–2.13)	1.02	0.99	0.99 (0.12–8.45)	
		Sample type (*p*-value = 0.64)	Blood	Ref.				
			Cervical swab	2.50 (−1.54–6.53)	1.92	0.21	12.15 (0.21–686.67)	
			Milk (individual)	0.34 (−2.15–2.83)	1.19	0.78	1.40 (0.12–16.95)	
			Pooled milk	0.61 (−2.46–3.67)	1.46	0.68	1.83 (0.09–39.16)	
Sheep	20	Intercept		−1.50 (−3.09–0.08)	0.74	0.06		τ^2^ = 0.58, *I*^2^ = 88.2%, R^2^ **=** 34.6%
		Test category	Direct	Ref.				
			Indirect	−1.51 (−3.17–0.16)	0.78	0.07	0.22 (0.04–1.17)	
		Sample type (*p*-value = 0.89)	Blood	Ref.				
			Milk (individual)	−0.74 (−3.60–2.12)	1.34	0.59	0.48 (0.03–8.33)	
			Pooled milk	0.40 (−4.14–4.95)	2.13	0.85	1.50 (0.02–141.18)	
			Semen	−0.59 (−3.02–1.84)	1.14	0.61	0.55 (0.05–6.30)	
Camels	17	Intercept		−1.82 (−2.36 to – ‐1.28)	0.25	<0.001		τ^2^ = 0.38, *I*^2^ = 82.5%, R^2^ **=** 70.0%
		Test category	Indirect	Ref.				
			Direct	1.98 (0.77–3.20)	0.56	**0.0037**	7.27 (2.16–24.54)	
		Sample type (*p*-value = 0.12)	Blood	Ref.				
			Milk (individual)	−1.33 (−4.14–1.48)	1.30	0.33	0.26 (0.02–4.41)	
			Pooled milk	−1.46 (−3.01–0.10)	0.72	0.06	0.23 (0.05–1.11)	
Animal model (domestic and wildlife)	137	Intercept		−1.26 (−1.95 to – ‐0.57)	0.35	0.0004		τ^2^ = 1.14, *I*^2^ = 97.3%, R^2^ **=** 13.3%
		Test category	Direct	Ref.				
			Indirect	−1.22 (−1.92 to – ‐0.51)	0.36	**0.0009**	0.30 (0.15–0.60)	
		Sample type (*p*-value = 0.14)	Blood	Ref.				
			Cervical swab	1.26 (−1.31–3.84)	1.30	0.33	3.53 (0.27–46.36)	
			Milk (individual)	−1.23 (−2.83–0.37)	0.81	0.13	0.29 (0.06–1.44)	
			Pooled milk	−1.69 (−3.44–0.05)	0.88	0.06	0.18 (0.03–1.05)	
			Semen	−0.83 (−3.15–1.48)	1.17	0.48	0.43 (0.04–4.40)	
		Host type	Domestic	Ref.				
			Wildlife	0.11 (−0.50–0.72)	0.31	0.73	1.11 (0.60–2.05)	
		Interaction (*p*-value = 0.06)	Indirect × milk	1.74 (−0.12–3.60)	0.94	0.07	5.68 (0.88–36.56)	
			Indirect × pooled milk	1.74 (−0.16–3.65)	0.96	0.07	5.71 (0.85–38.43)	

Ref.: reference, SE: standard error, CI: confidence intervals.

⁎ Other: (community, slaughterhouse workers, HIV seropositive patients).

† Studies with missing data were dropped.

‡ The intercept for the “study year” variable refers to a study that was conducted in the year 2000.

The models for cattle, goats, and sheep found no significant associations with the test category, sample type, or study end year (all *p*-value > 0.05), and models explained less than 35% of the heterogeneity. There was also a high residual heterogeneity observed in all the models (*I*^2^ > 80%).

The human model showed no considerable predictor of between-study heterogeneity, and a high proportion of unaccounted heterogeneity was observed in the final model (τ^2^ = 2.62, *I*^2^ = 99.4%, R^2^ = 0.0%).

The combined animal model revealed that direct tests had 3.37 (95% CI: 1.66–6.85, *p*-value = 0.0009) times higher odds of determining positive cases than indirect tests. However, this effect was reduced due to potential interaction between sample type and diagnostic tests (overall interaction *p*-value = 0.06) (Table 3). Lastly, when adjusted for the type of sample and diagnostic test, there was interestingly no clear difference in the prevalence estimates between domestic and wild animals (*p*-value = 0.73).

### 3.4 Bipartite network results

**Fig. 2:**
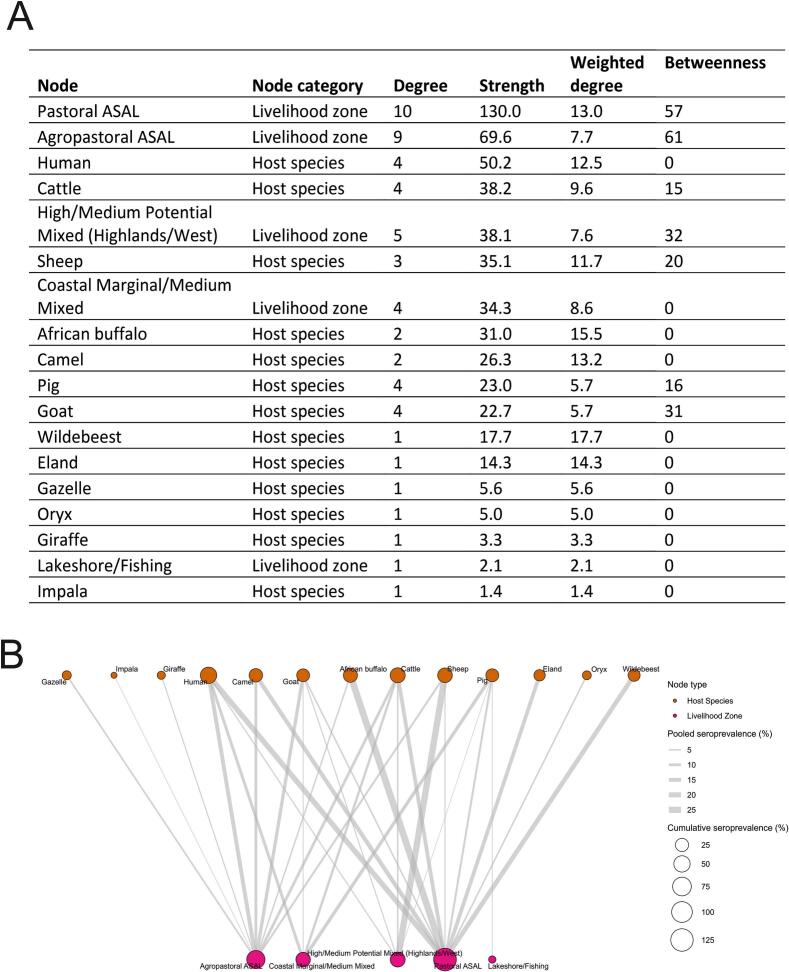
**A.** A summary of node-level metrics for the bipartite host-livelihood zone network analysis with edges weighted by the pooled seroprevalence for each connection. Only indirect tests on blood samples were admitted to these analyses to reduce potential sources of heterogeneity. **B.** Bipartite network linking hosts to livelihood zones, with the thickness of their connections representing seroprevalence pooled by meta-analysis. Node size represents the cumulative seroprevalence of all its connections (node strength).

The host-livelihood zone bipartite network analysis revealed that the pastoral arid and semi-arid lands (ASAL) were the most connected zone (degree = 10) and had connections with the highest cumulative seroprevalence (strength = 130.0). This was followed by the Agropastoral ASAL zone which was the most central hub that connects various hosts and livelihood zones in Kenya's brucellosis landscape (betweenness = 61). The Lakeshore/Fishing zone was the most peripheral livelihood zone (degree = 1) (Fig. 2).

Regarding host nodes, humans showed the highest cumulative seroprevalence (strength = 50.2), and a high average seroprevalence (weighted degree = 12.5). However, humans did not act as important linkages between other network nodes (betweenness = 0).

Domestic animals were important nodes, with cattle showing the highest cumulative seroprevalence (strength = 38.2) and connecting four other nodes (degree = 4), thereby bearing the highest animal brucellosis prevalence. However, goats exhibited the highest betweenness centrality of all hosts (betweenness = 31), despite their lower cumulative (22.7) and average (5.7) seroprevalence. Sheep were the second most important domestic connectors observed in the network (betweenness = 20).

Wildlife species exhibited a considerable cumulative seroprevalence, especially buffaloes (strength = 31.0) and wildebeest (strength = 17.7). However, all wildlife showed a betweenness of 0 (Fig. 2).

The analysis of theoretically possible host-livelihood zone combinations revealed that all studies included in the network analysis covered 29/65 possible combinations, which gives a 44.6% brucellosis research coverage and 55.4% unstudied theoretical combinations (a proxy for research gaps). Other network-level metrics were a simple bipartite density of 0.19 (contains within-node pairs), a component of 1, unweighted diameter of 5 and unweighted average pathlength of 2.25 (Fig. 2).

A confirmatory multivariable model was fitted on the network data to reveal that hosts from the Agropastoral ASAL regions had 3.18 (95% CI: 1.81–5.58, *p*-value <0.0001) times higher odds of being seropositive than those from High/Medium potential mixed (Highlands/West) zones. The final model controlled for host category, i.e., human, domestic or wildlife hosts (τ^2^ = 0.82, *I*^2^ = 95.2%, R^2^ = 14.9%).

## 4 Discussion

We systematically combined 55 years of brucellosis research comprising 20,944 humans, 57,340 domestic species, 727 wildlife and 235 vector samples from various areas in Kenya for the first time. Key findings were considerable pooled seroprevalence estimates in livestock and humans, albeit with high between-study heterogeneity. Meta-regression analyses revealed that direct detection methods had higher odds of reporting positive results than indirect assays in animals generally, and in camel studies in particular. A bipartite network of hosts and locations sampled showed a sparse but connected brucellosis research network, with the highest brucellosis presence being from pastoral and agropastoral ASAL areas and included a few key hosts (humans, cattle, sheep, goats and camels). Many other host-location combinations such as the lakeshore/fishing areas and wildlife species received less attention.

There was an increase in brucellosis research with time, especially in the last two decades. This is likely as a result of prioritisation of brucellosis as a key zoonosis, and later the development of a national strategy for brucellosis prevention and control [10], [17]. Most studies were also conducted in the pastoral/agropastoral and high/medium potential counties such as Garissa, Nairobi, Kajiado, Narok, Isiolo, and Marsabit, while many counties in the western (Siaya, Vihiga, Homa Bay, Bomet, Nyamira) and central regions (Tharaka, Embu) had no studies altogether.

Our quantitative analysis revealed domestic animals and humans to have considerable seroprevalence estimates using indirect serological tests on blood samples from studies with probability sampling methods (7.1% in cattle, 9.7% in camels, 11.4% in goats, 5.2% in sheep, and 9.8% in apparently healthy humans). These estimates were lower than those from India (16.6% in cattle using probability sampling) [18]. Kenya therefore has a considerable brucellosis presence that requires continued investment in disease control.

We observed very high heterogeneity in all estimates, with most models partially explaining the heterogeneity and some models (humans and goats) explaining none altogether (R^2^ = 0.0%). Other factors such as herd characteristics (sex, age, size, etc.), animal production system (intensive, semi-intensive, extensive, etc.), vaccination status (performed in some private ranches), animal movement patterns, finer diagnostic test characteristics, etc. could therefore play a role in the heterogeneity of brucellosis studies in the country. However, these details were not always available in the included publications or were not included in our meta-regression due to the few study numbers. Additionally, the predominance of non-probability sampling and the limited use of sample size calculations can introduce selection bias and limit the generalisability of results to the population [19], including our meta-analytic estimates in this review. This reliance on non-probability and mixed approaches could be as a result of logistical challenges in field epidemiological studies that make rigorous probability sampling methods impractical or overly expensive. When stratified by sampling methodology, many subgroup analyses had limited data, wide confidence intervals, and several estimates were even based on single studies rather than meta-analytic pooling from multiple studies which limits direct comparisons between sampling approaches. The overall point estimates for each species varied slightly between sampling approach, but confidence intervals overlapped in all instances. However, the predominance of non-probability sampling means these estimates require cautious interpretation. Future studies should prioritise probability-based sampling designs where possible and report sample size calculation to improve the quality and comparability of prevalence estimates.

Direct diagnostic tests (e.g., PCR and culture) had higher odds of determining positive cases in animals, and in camel data specifically. The use of PCR and culture on clinically suspicious samples or only serologically positive samples could explain this finding, although PCR is increasingly being applied in brucellosis diagnosis in the country. This selective application enables bacterial detection and species determination with minimal resources. The pooled estimates by PCR may therefore not represent the true prevalence due to a lack of the same randomly sampled denominator as the serological tests. Using PCR offers high diagnostic accuracy, early detection during incubation and acute phases of clinical progression, differentiation from vaccine strains, monitoring treatment responses for relapses, testing of matrices with no simple serological tests (e.g., milk from some domestic species, fermented milk such as *mala* and *mursik*), and species identification which can elucidate the transmission dynamics [20], [21], [22]. Despite this, serological testing remains essential for brucellosis diagnosis in resource-limited settings due to its accessibility and cost-effectiveness. Future epidemiological studies in Kenya that apply both serological and PCR testing to the same sampled population will help distinguish active infections from past ones and reduce the bias brought about by selective sampling frames or indeed diagnostic test usage.

There were several key outputs from the network analysis. The pastoral and agropastoral regions bear the highest brucellosis estimates and are the most consequential transmission zones in Kenya from the high strength, betweenness centrality, and meta-regression odds estimates found. These zones also connect the majority of the studied host species. Most of Kenya's brucellosis research is focussed on these areas, and rightly so due to the large livestock numbers, close human-animal contact, risky practices such as drinking raw milk, limited access to health care and veterinary extension services, and sharing of (unprotected) water sources between domestic and wild animals [23], [24]. Prioritising ASAL regions of Kenya is therefore required for adequate brucellosis control. However, studies should include less-studied zones such as lakeshore/fishing (k = 1), coastal and high potential areas which showed considerable disease presence.

Humans had the highest network strength (cumulative seroprevalence) of all hosts, demonstrating the immediate public health concern. Human prevalence also likely varies by geographical zone as humans had the second highest estimates in the meta-analysis results which did not consider location. Cattle had the highest disease presence in domestic animals, indicating the need for bovine brucellosis control in the country. Goats had the highest betweenness centrality of host species and therefore connect otherwise isolated zones (e.g., Coastal Marginal/Medium Mixed) to pastoral zones with higher brucellosis presence despite their lower cumulative seroprevalence. This bridging role suggests that even moderate goat seroprevalence estimates may have disproportionate epidemiological consequences if goat movement facilitates spread of brucellosis to other zones or to other susceptible hosts such as cattle. Molecular studies in the country have indeed identified *B. melitensis* commonly associated with caprine and ovine brucellosis in other hosts (cattle, buffaloes, giraffes, camels), and *B. abortus* in goats and other non-bovine hosts which suggests the spread of animal brucellosis between species in mixed livestock production systems [25], [26], [27], [28], [29], [30], [31]. The extent to which goat trade networks contribute to spatial spread is unclear from our seroprevalence-based network and requires investigation through livestock movement mapping and molecular epidemiology (strain typing, whole-genome sequencing) to elucidate transmission pathways. No species had the highest strength (cumulative seroprevalence) and highest betweenness centrality simultaneously, with high seroprevalence species (humans and cattle) being confined to the well-connected pastoral zones, and bridging species with high betweenness (goats, sheep, and pigs) with relatively lower seroprevalence. The spread of brucellosis between zones may therefore be limited perhaps due to geographic barriers, farming practices or even varying species-specific livestock movements/trade networks or animal migration between zones. Our network was constructed from seroprevalence studies rather than direct observations of animal movement or *Brucella* transmission events and is therefore more reflective of research patterns than biological transmission dynamics. These findings can thus not adequately inform zone-specific control strategies as we cannot determine whether targeted interventions in high-risk zones would remain effective considering potential reintroduction from connected zones from seroprevalence data alone. However, the concentration of seropositive animals in pastoral zones suggests that even if inter-zonal transmission occurs, ASAL-focused interventions targeting cattle would reduce the largest disease reservoirs and likely decrease spill-over to humans and less-studied zones. Mapping livestock movements and animal migration routes at a national level and integrating genomic characterisation of isolates from various zones to demonstrate shared strains in circulation will further enrich zone-specific control measures. Current policy for prevention, control and diagnosis should consider the spatial heterogeneity of brucellosis research and operationalise zone-specific measures [17], [32]. These could include comprehensive cattle vaccination in pastoral Kenya, multi-species programs in agropastoral areas, and human brucellosis screening of febrile cases.

Wildlife species demonstrated considerable seroprevalence where studied, but all had a betweenness of 0. This suggests an exclusion of wildlife from animal brucellosis research in the country. There also existed very few prevalence studies on wildlife in comparison to humans and domestic animals, which indicates that limited attention is given to wildlife species in brucellosis research. The detection of brucellae in biting flies (keds) collected from dogs in one study requires further investigation on mechanical transmission of brucellosis, especially from under-researched hosts such as canines [33]. A One Health approach is required to elucidate the role of wildlife in brucellosis epidemiology in the country, especially in regions with close domestic animal-wildlife interaction to avoid research coverage blind spots. The meta-regression found no significant difference in domestic and wildlife seroprevalence, even though this could be from the lack of statistical power given the few wildlife studies. The comparable or even higher seroprevalence indicates that wildlife could harbour brucellae and transmit at domestic-wildlife interfaces as evidenced by *B. melitensis* detection from buffalo and giraffe samples [27]. However, the directionality of transmission can only be elucidated by molecular epidemiological studies.

Several geographical areas and hosts are unexplored as shown by the estimated 55.4% research gap despite more than half a century of brucellosis research. Examples of this gap are the Lakeshore/Fishing zone which is severely understudied (only 1 dated study on pigs from Kisumu, *n* = 23) [34], which means we cannot adequately assess brucellosis epidemiology in communities at the lake basin. The Coastal Marginal/Medium Mixed zone also had only 7 studies on 4 species. Kenya's national prevention and control strategy includes measures such as pasteurisation/boiling, culling, biosafety, livestock vaccination (S19 and Rev1 vaccine strains for cattle and small ruminants, respectively), quarantine and movement control [17], and their adoption in priority areas and hosts is recommended.

Our study had several limitations. We observed high heterogeneity in the meta-analytic estimates and meta-regression analyses that would not be explained by the moderators we explored, which limits the interpretability of our estimates. We also combined the various livelihood zones into a few manageable categories, which may not fully capture the unique characteristics of livelihood zones that can influence disease spread. We also excluded direct detection methods in our network analyses, which likely underestimated the role of active infections in the brucellosis research network we constructed.

In conclusion, we observed considerable brucellosis presence in humans and key livestock in Kenya. Pastoral and agropastoral ASAL zones are the most consequential zones in brucellosis research, but the published evidence has considerable geographic gaps (e.g., lakeshore/fishing communities) and under-represents wildlife. Future epidemiological studies will have outputs that are more representative of the population when probabilistic sampling approaches are utilised. Lastly, adopting a One Health framework that includes wildlife is required in future epidemiological studies in the country for adequate brucellosis risk assessment.

## CRediT authorship contribution statement

**Martin Wainaina:** Writing – review & editing, Writing – original draft, Visualization, Validation, Supervision, Resources, Project administration, Methodology, Investigation, Formal analysis, Conceptualization. **Joseph Samuel Kimatu:** Writing – review & editing, Validation, Methodology, Investigation. **Benson Rukwaro:** Writing – review & editing, Validation, Methodology, Investigation. **Elizabeth Anne Jessie Cook:** Writing – review & editing, Validation, Supervision, Resources, Project administration, Methodology, Investigation.

## Funding

This work was supported by 10.13039/501100008751the German Federal Institute for Risk Assessment (BfR), the Federal Minister of Agriculture, Food and Regional Identity (BMLEH) as part of the research project “KI- & Daten-Akzelerator (KIDA)” of project number 28KIDA004, and the CGIAR Sustainable Animal and Aquatic Foods (SAAF) Science Program. CGIAR research is supported by contributions to the CGIAR Trust Fund. The funders had no role in study design, data collection and analysis, decision to publish, or preparation of the manuscript.

## Declaration of competing interest

The authors declare that they have no known competing financial interests or personal relationships that could have appeared to influence the work reported in this paper.

## Data Availability

All data relating to the present study are available in this manuscript and supplementary files.

## Appendix A Supplementary data

Supplementary material 1 Image 1

Supplementary material 2 Image 2
