# Supplementary material for: Epidemiology of human and animal brucellosis in Kenya: A One Health meta-regression and network analysis
Source: One Health. 2026 Mar 18;22:101390. doi: 10.1016/j.onehlt.2026.101390 (PMC13049966; doi:10.1016/j.onehlt.2026.101390)
Supplement: Supplementary file 1 — Supplementary material 1 [file mmc1.docx]

**Epidemiology of human and animal brucellosis in Kenya: A One Health meta-regression and network analysis**

Martin Wainaina, Joseph Samuel Kimatu, Benson Rukwaro, Elizabeth Anne Jessie Cook

**Table of contents**

[**Literature searches** 3](#_Toc224822347)

[**PRISMA flowchart** 5](#_Toc224822348)

[**Forest plots (All Studies)** 6](#_Toc224822349)

[**Cattle forest plot** 6](#_Toc224822350)

[**Human forest plot** 7](#_Toc224822351)

[**Goat forest plot** 8](#_Toc224822352)

[**Sheep forest plot** 9](#_Toc224822353)

[**Camel forest plot** 10](#_Toc224822354)

[**Forest plots (Studies with probability sampling methods)** 11](#_Toc224822355)

[**Cattle forest plot – probability sampling** 11](#_Toc224822356)

[**Human forest plot – probability sampling** 12](#_Toc224822357)

[**Goat forest plot – probability sampling** 13](#_Toc224822358)

[**Sheep forest plot – probability sampling** 13](#_Toc224822359)

[**Camel forest plot – probability sampling** 14](#_Toc224822360)

[**Forest plots (Studies with non-probability sampling methods)** 15](#_Toc224822361)

[**Cattle forest plot – non-probability sampling** 15](#_Toc224822362)

[**Human forest plot – non-probability sampling** 16](#_Toc224822363)

[**Goat forest plot – non-probability sampling** 17](#_Toc224822364)

[**Sheep forest plot – non-probability sampling** 17](#_Toc224822365)

[**Camel forest plot – non-probability sampling** 18](#_Toc224822366)

[**Influence analyses (All studies)** 19](#_Toc224822367)

[**Human influence plots** 19](#_Toc224822368)

[**Camel influence plots** 20](#_Toc224822369)

[**Sheep influence plots** 21](#_Toc224822370)

[**Goat influence plots** 22](#_Toc224822371)

[**Cattle influence plots** 23](#_Toc224822372)

[**Livelihood zones** 24](#_Toc224822373)

[**Original livelihood zones** 24](#_Toc224822374)

[**Combined zones map** 25](#_Toc224822375)

[**Network analyses** 26](#_Toc224822376)

[**Edge weights** 26](#_Toc224822377)

[**Theoretical research gaps** 28](#_Toc224822378)

[**Study characteristics table** 29](#_Toc224822379)

[**Bibliography** 33](#_Toc224822380)

# **Literature searches**

Searches were performed on 11^th^ July 2025 using the following search terms:

((brucellosis OR brucella OR brucel* OR "Malta fever" OR "Mediterranean fever" OR "undulant fever") AND (prevalence OR incidence OR risk OR control OR prevention) AND (Kenya) AND (human OR livestock OR wildlife OR domestic OR rodent OR ruminant OR (cattle OR bovine) OR (camel OR dromedary) OR (sheep OR ovine) OR (goat OR caprine) OR (pig OR swine)))

Supplementary Table 1: Search strings used for literature searches in the five databases for this systematic review

| **Databases** | **Search string** | **Database hits** |
| --- | --- | --- |
| PubMed | (((brucellosis OR brucella OR brucel* OR "Malta fever" OR "Mediterranean fever" OR "undulant fever")  AND  (prevalence OR incidence OR risk OR control OR prevention))  AND  (Kenya))  AND  (human OR livestock OR wildlife OR domestic OR rodent OR ruminant OR (cattle OR bovine) OR (camel OR dromedary) OR (sheep OR ovine) OR (goat OR caprine) OR (pig OR swine)) | 117 |
| Scopus | All fields(brucellosis OR brucella OR brucel* OR "Malta fever" OR "Mediterranean fever" OR "undulant fever")  AND  All fields(Kenya)  AND  All fields (prevalence OR incidence OR risk OR control OR prevention)  AND  All fields (human OR livestock OR wildlife OR domestic OR rodent OR ruminant OR (cattle OR bovine) OR (camel OR dromedary) OR (sheep OR ovine) OR (goat OR caprine) OR (pig OR swine)) | 2620 |
| Embase | ('brucellosis'/exp OR brucellosis OR 'brucella'/exp OR brucella OR brucel* OR 'malta fever'/exp OR 'malta fever' OR 'mediterranean fever'/exp OR 'mediterranean fever' OR 'undulant fever'/exp OR 'undulant fever')  AND  ('prevalence'/exp OR prevalence OR 'incidence'/exp OR incidence OR 'risk'/exp OR risk OR 'control'/exp OR control OR 'prevention'/exp OR prevention)  AND  ('kenya'/exp OR kenya)  AND  ('human'/exp OR human OR 'livestock'/exp OR livestock OR 'wildlife'/exp OR wildlife OR domestic OR 'rodent'/exp OR rodent OR 'ruminant'/exp OR ruminant OR 'cattle'/exp OR cattle OR 'bovine'/exp OR bovine OR 'camel'/exp OR camel OR 'dromedary'/exp OR dromedary OR 'sheep'/exp OR sheep OR 'ovine'/exp OR ovine OR 'goat'/exp OR goat OR 'caprine'/exp OR caprine OR 'pig'/exp OR pig OR 'swine'/exp OR swine) | 154 |
| Africa Journals Online | Google Scholar search was:  site:www.ajol.info ((brucellosis OR brucella OR brucel* OR "Malta fever" OR "Mediterranean fever" OR "undulant fever") AND (prevalence OR incidence OR risk OR control OR prevention) AND (Kenya) AND (human OR livestock OR wildlife OR domestic OR rodent OR ruminant OR (cattle OR bovine) OR (camel OR dromedary) OR (sheep OR ovine) OR (goat OR caprine) OR (pig OR swine))) | 145 |
| Web of Science | brucellosis OR brucella OR brucel* OR "Malta fever" OR "Mediterranean fever" OR "undulant fever" (All Fields)  AND  Kenya (All Fields)  AND  prevalence OR incidence OR risk OR control OR prevention (All Fields)  AND  human OR livestock OR wildlife OR domestic OR rodent OR ruminant OR (cattle OR bovine) OR (camel OR dromedary) OR (sheep OR ovine) OR (goat OR caprine) OR (pig OR swine) (All Fields) | 148 |
| **Total** |  | **3184** |

# **PRISMA flowchart**


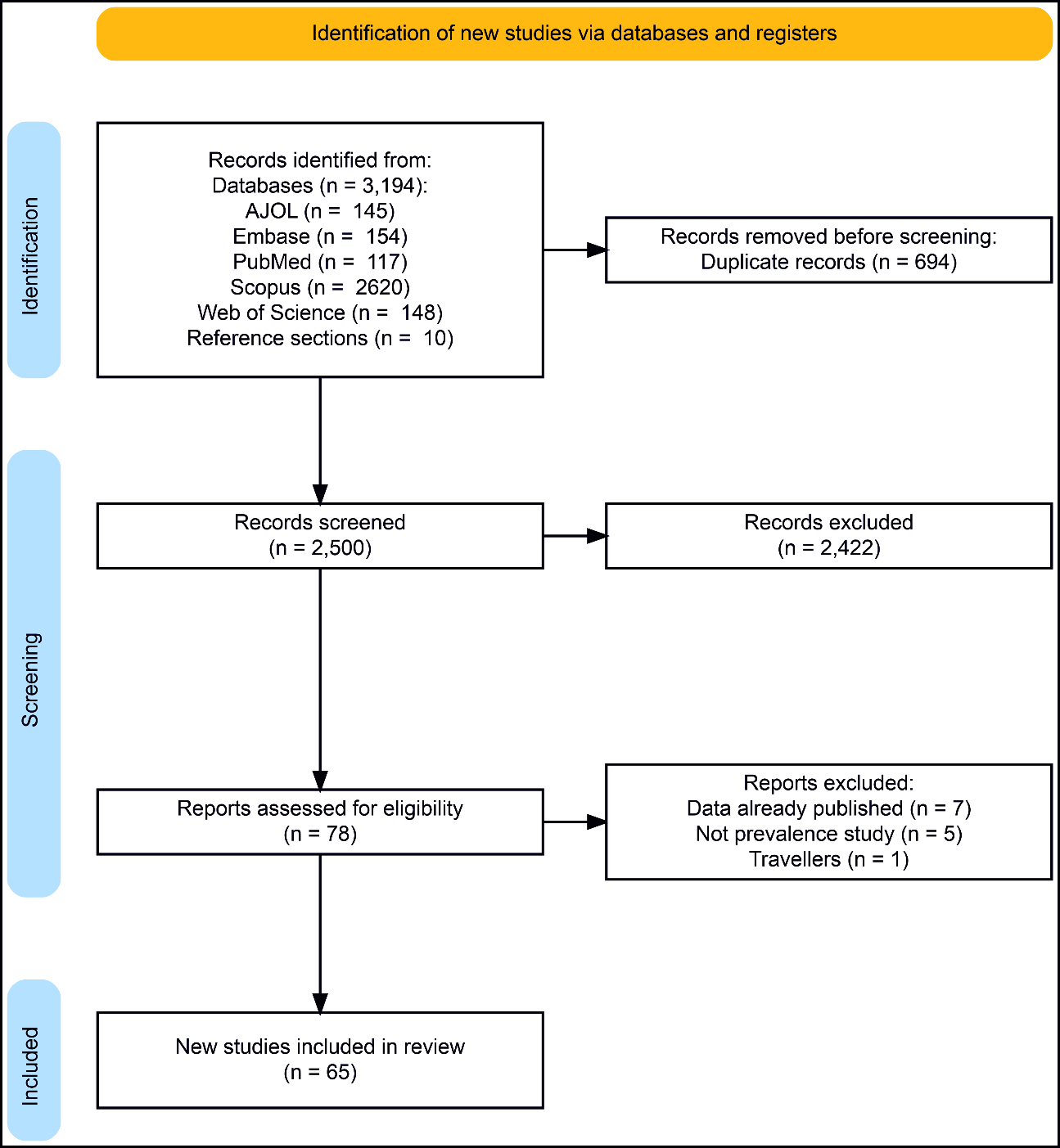


*Figure 1: PRISMA flowchart showing the study selection process*

AJOL: African Journals online

# **Forest plots (All Studies)**

## **Cattle forest plot**


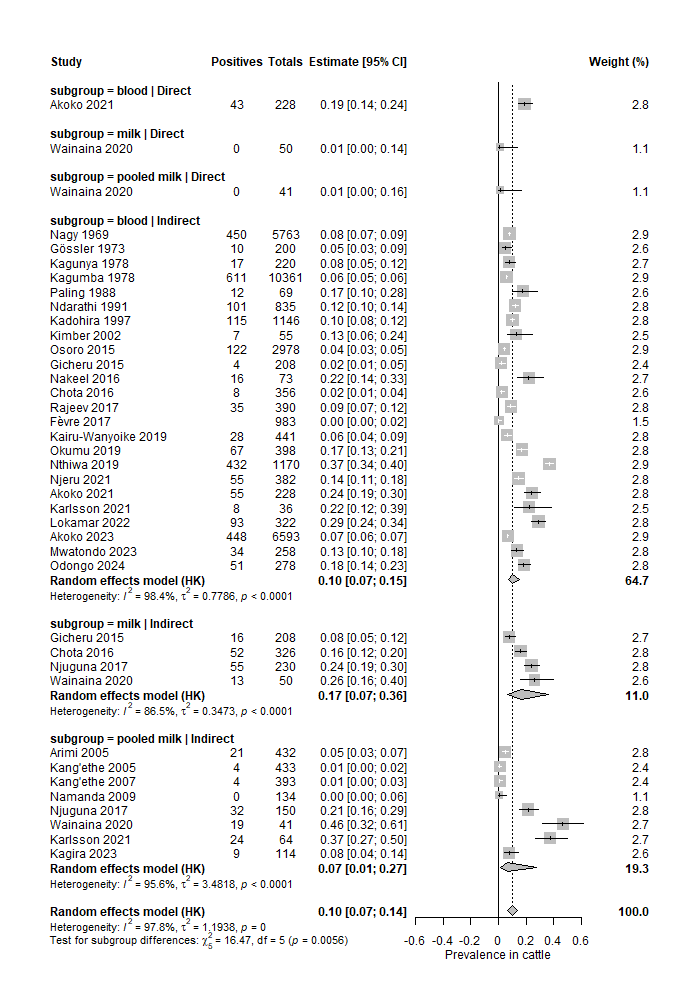


*Figure 2: Forest plot of studies investigating brucellosis in cattle. Results of serological tests such as CFT that utilise B. abortus (S99 strain) were preferred as recommended by the WOAH to enable comparability.*

## **Human forest plot**


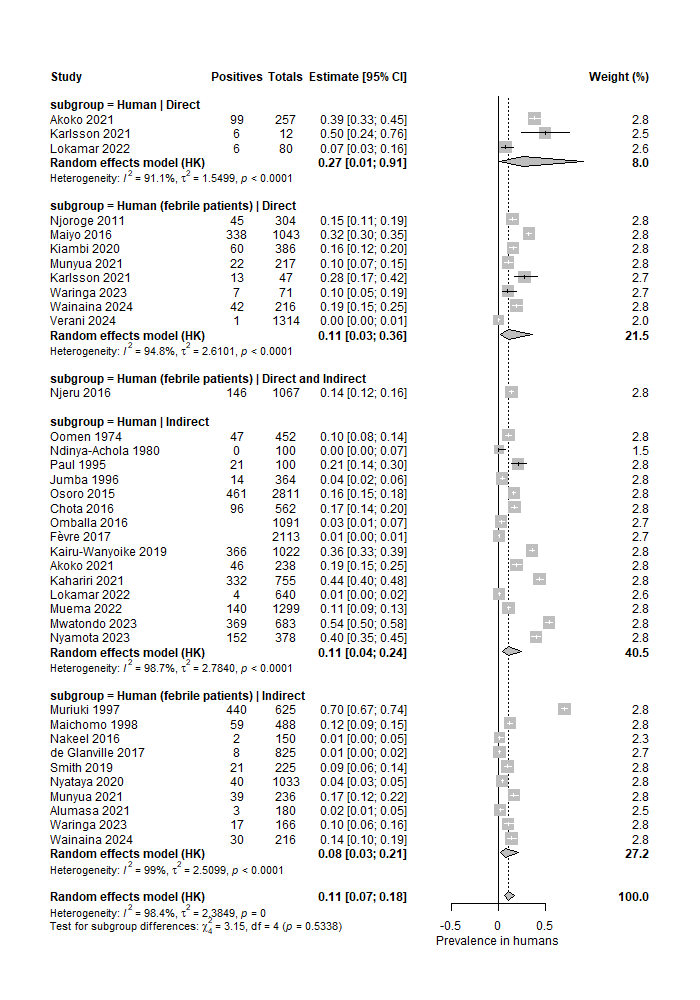


*Figure 3: Forest plot of studies investigating brucellosis in humans*

## **Goat forest plot**


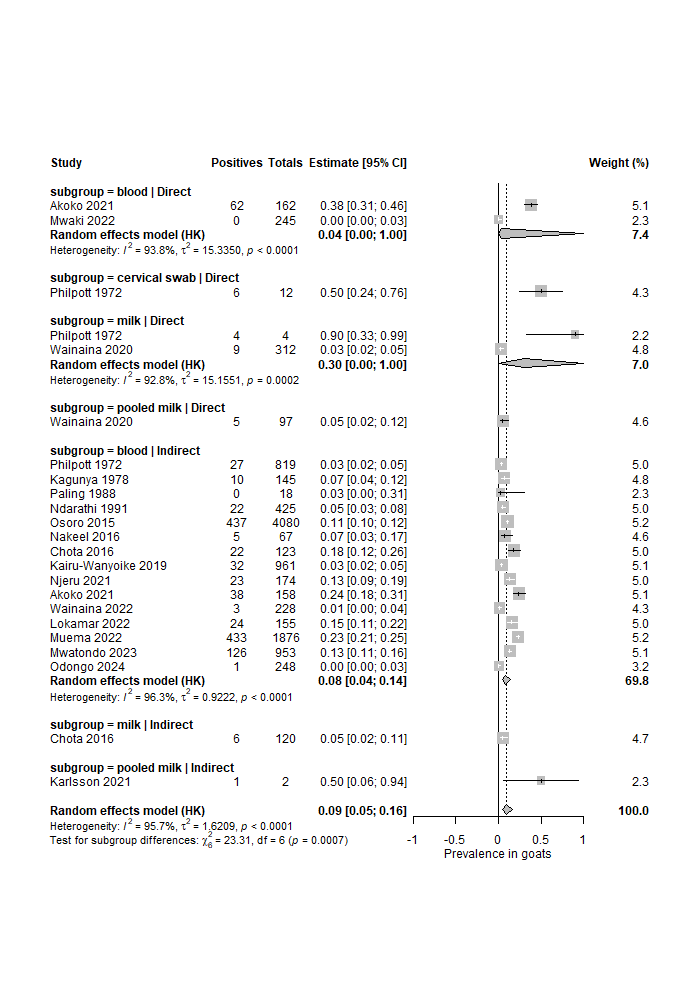


*Figure 4: Forest plot of studies investigating brucellosis in goats*

## **Sheep forest plot**


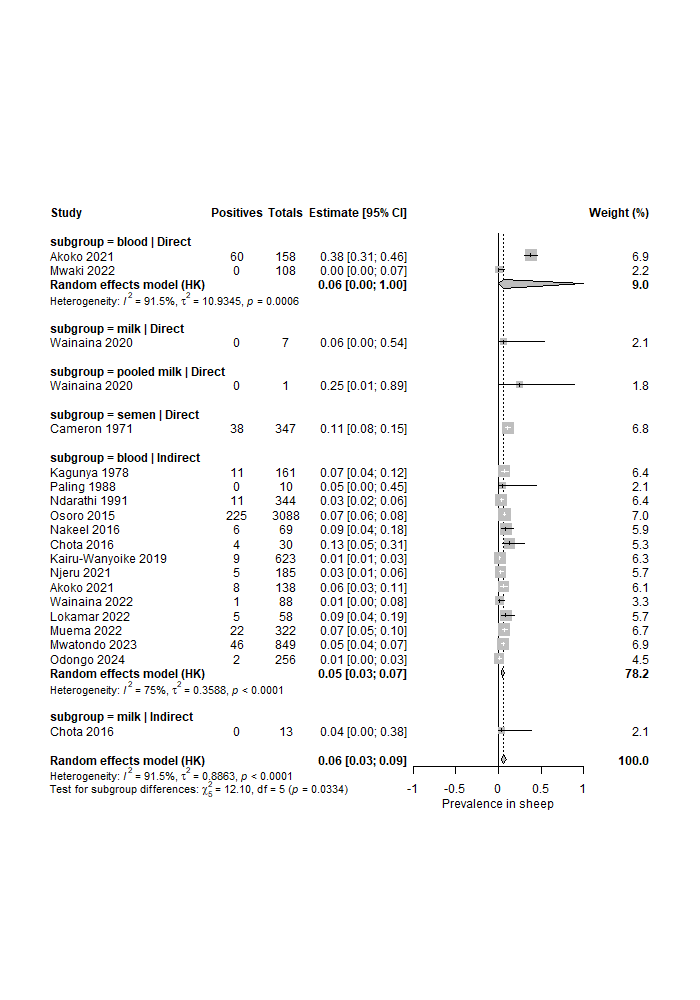


*Figure 5: Forest plot of studies investigating brucellosis in sheep*

## **Camel forest plot**


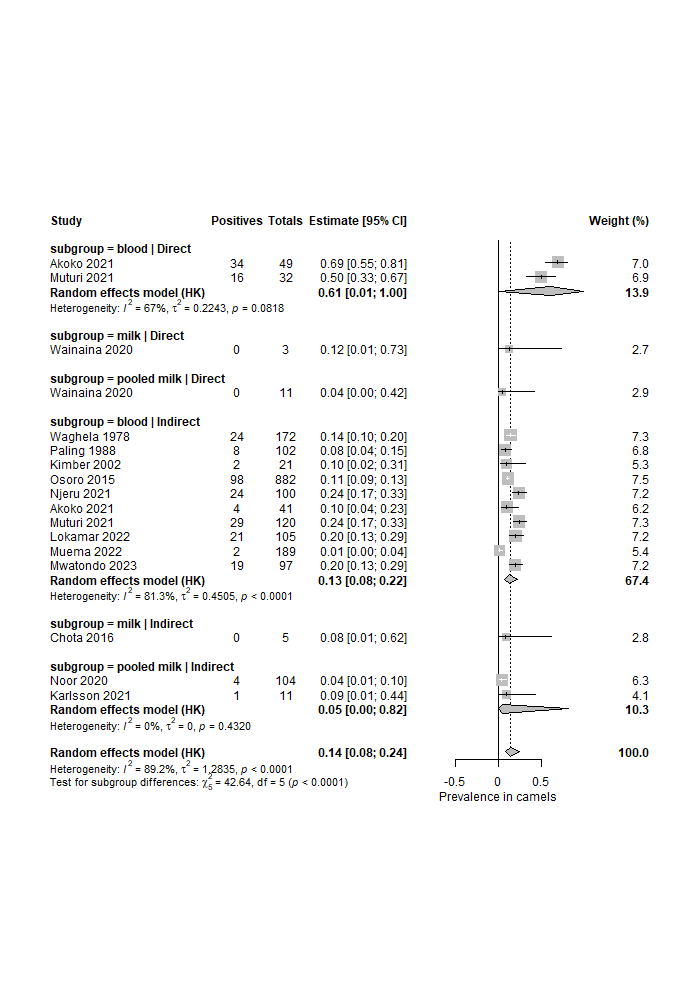


*Figure 6: Forest plot of studies investigating brucellosis in camels*

# **Forest plots (Studies with probability sampling methods)**

## **Cattle forest plot – probability sampling**


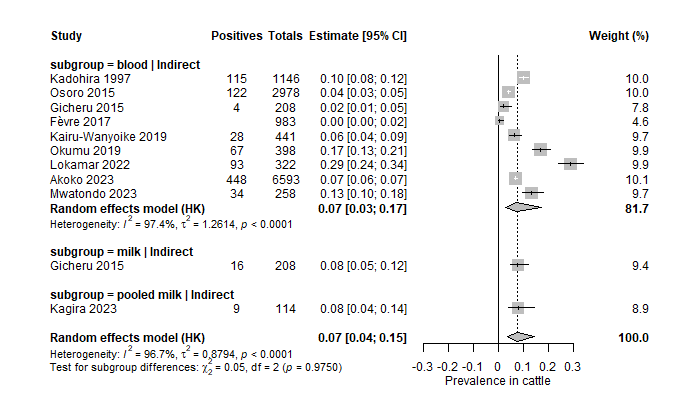


Figure 7: Forest plot of studies investigating brucellosis in cattle with probability sampling methods

## **Human forest plot – probability sampling**


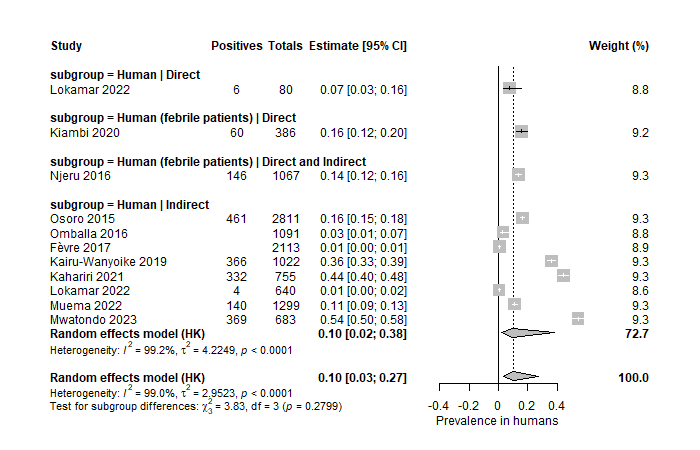


Figure 8: Forest plot of studies investigating brucellosis in humans with probability sampling methods

## **Goat forest plot – probability sampling**


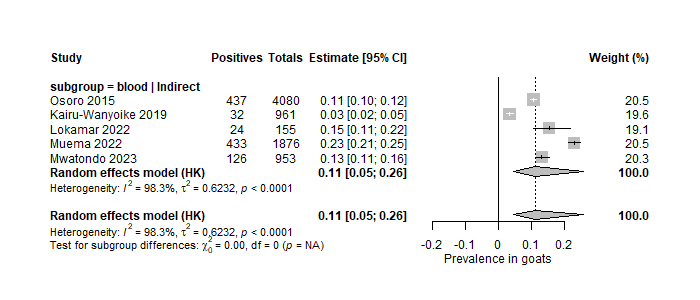


Figure 9: Forest plot of studies investigating brucellosis in goats with probability sampling methods

## **Sheep forest plot – probability sampling**


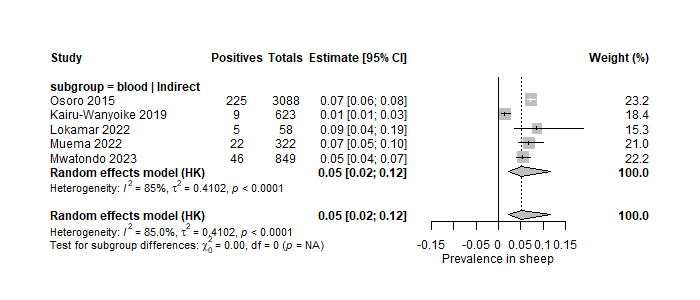


Figure 10: Forest plot of studies investigating brucellosis in sheep with probability sampling methods

## **Camel forest plot – probability sampling**


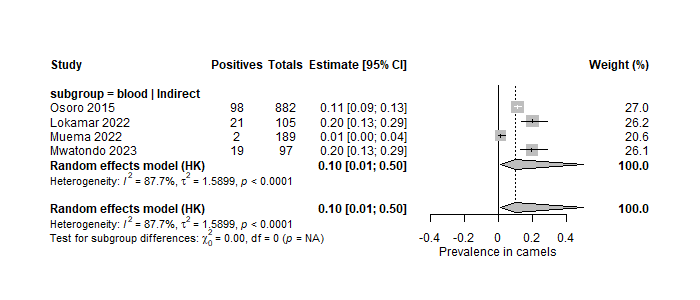


Figure 11: Forest plot of studies investigating brucellosis in camels with probability sampling methods

# **Forest plots (Studies with non-probability sampling methods)**

## **Cattle forest plot – non-probability sampling**


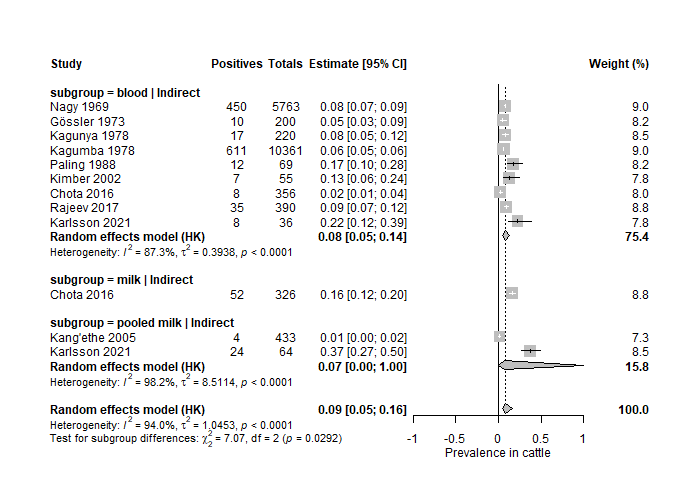


Figure 12: Forest plot of studies investigating brucellosis in cattle with non-probability sampling methods

## **Human forest plot – non-probability sampling**


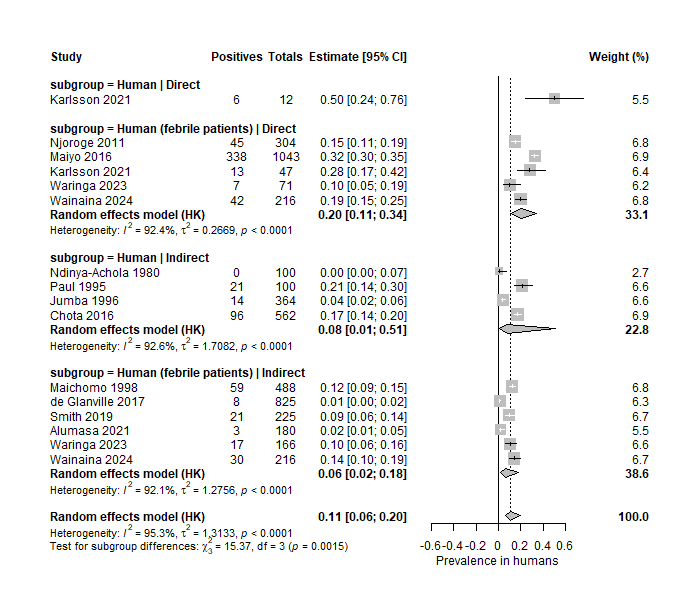


Figure 13: Forest plot of studies investigating brucellosis in humans with non-probability sampling methods

## **Goat forest plot – non-probability sampling**


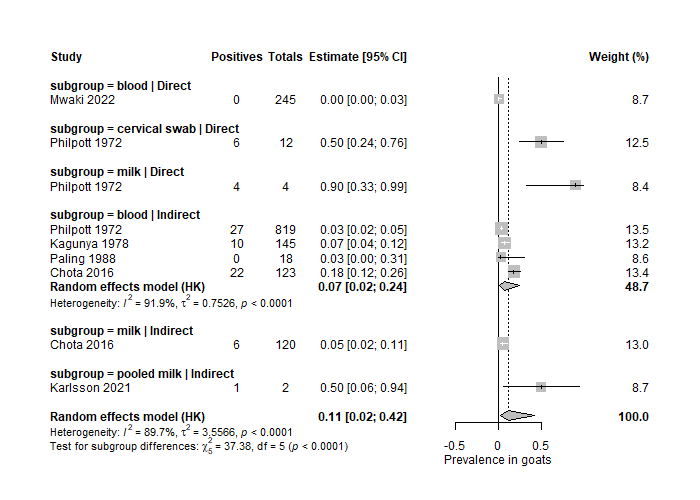


Figure 14: Forest plot of studies investigating brucellosis in goats with non-probability sampling methods

## **Sheep forest plot – non-probability sampling**


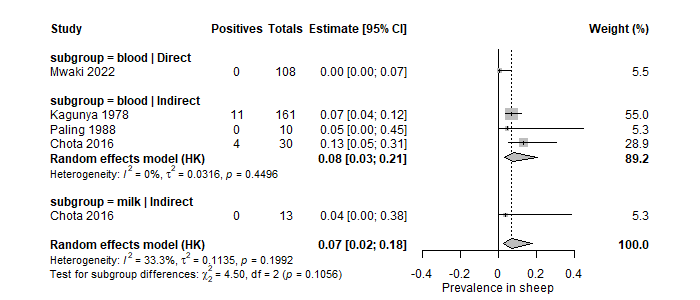


Figure 15: Forest plot of studies investigating brucellosis in sheep with non-probability sampling methods

## **Camel forest plot – non-probability sampling**


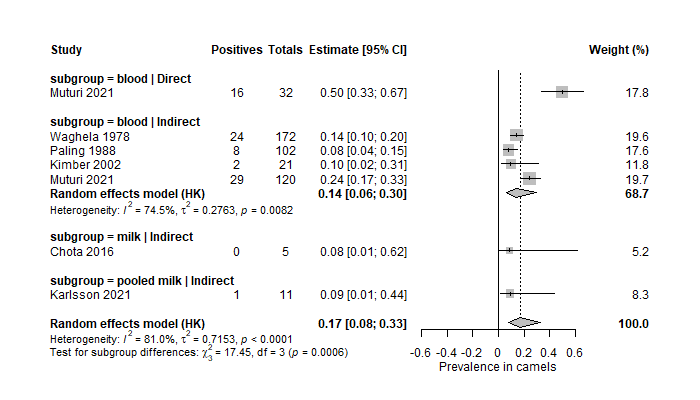


Figure 16: Forest plot of studies investigating brucellosis in camels with non-probability sampling methods

# **Influence analyses (All studies)**

## **Human influence plots**


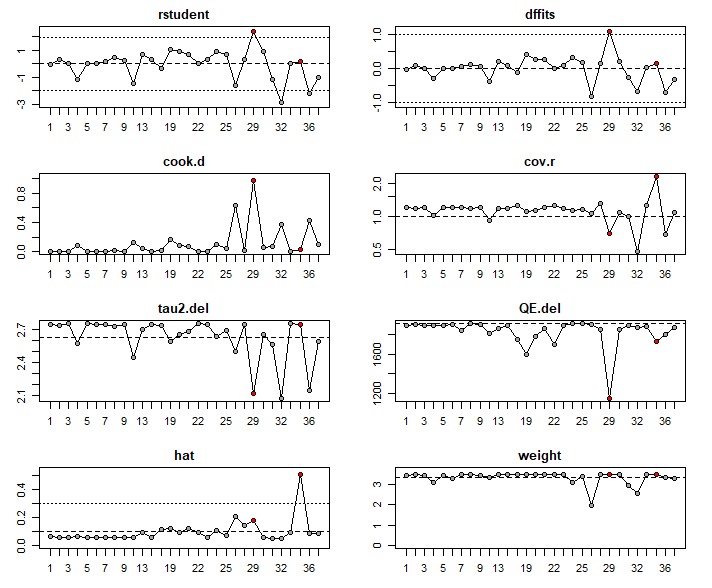


## **Camel influence plots**


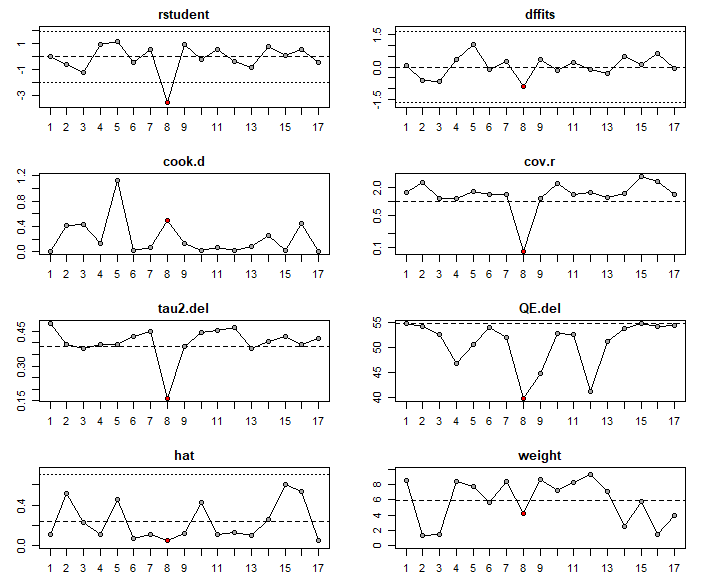


## **Sheep influence plots**


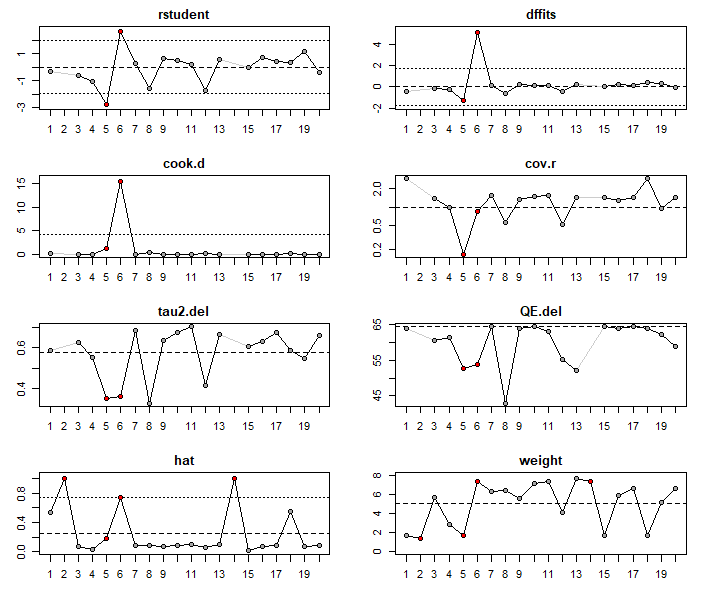


## **Goat influence plots**


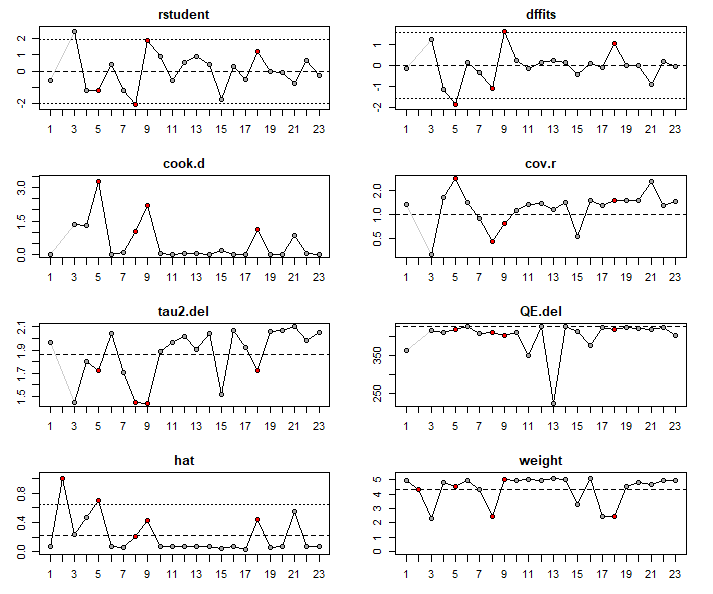


## **Cattle influence plots**


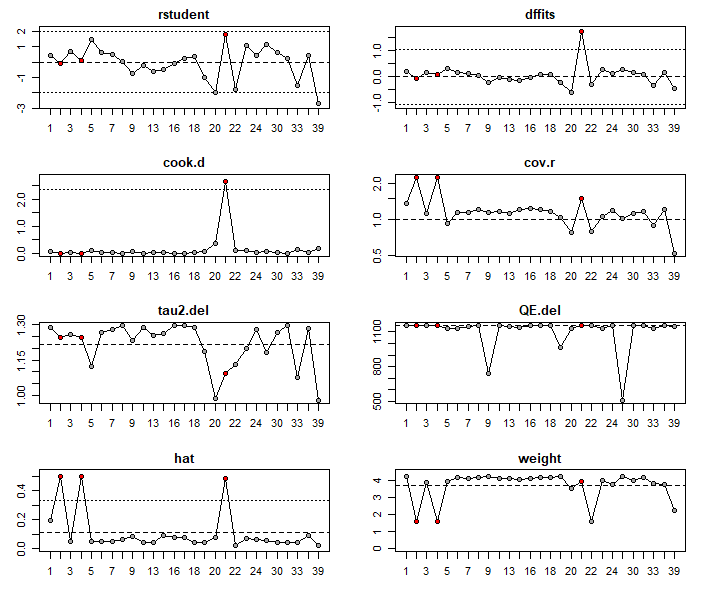


# **Livelihood zones**

## **Original livelihood zones**

*Table 1: Combined livelihood zones for Kenya used in the network analysis. The livelihood zones are defined by the Famine Early Warning System Network (FEWS NET).* [*https://fews.net/east-africa/kenya/livelihood-zone-map/march-2011*](https://fews.net/east-africa/kenya/livelihood-zone-map/march-2011)

| **Combined zones** | **FEWS Livelihood zones** |
| --- | --- |
| **Agropastoral ASAL** | KE03 - Northwestern Agropastoral Zone |
|  | KE06 - Marsabit Marginal Mixed Farming Zone |
|  | KE07 - Northeastern Agropastoral Zone |
|  | KE18 - Southern Agropastoral Zone |
|  | KE24 - Western Agropastoral Zone |
| **Coastal Marginal/Medium Mixed** | KE12 - Coastal Medium Potential Farming Zone |
|  | KE13 - Coastal Marginal Agricultural Mixed Farming Zone |
|  | KE16 - Southeastern Marginal Mixed Farming Zone |
| **High/Medium Potential Mixed (Highlands/West)** | KE17 - Southeastern Medium Potential, Mixed Farming Zone |
|  | KE19 - Central Highlands, High Potential Zone |
|  | KE20 - Western Medium Potential Zone |
|  | KE21 - Western High Potential Zone |
| **Lakeshore/Fishing** | KE04 - Lake Turkana Fishing Zone |
|  | KE22 - Western Lakeshore Marginal Mixed Farming Zone |
|  | KE23 - Lake Victoria Fishing Zone |
| **Pastoral ASAL** | KE01 - Northwestern Pastoral Zone |
|  | KE05 - Northern Pastoral Zone |
|  | KE09 - Northeastern Pastoral Zone |
|  | KE10 - Eastern Pastoral Zone |
|  | KE11 - Southeastern Pastoral Zone |
|  | KE15 - Southern Pastoral Zone |
|  | KE02 - Turkwell Riverine Zone |
|  | KE08 - Mandera Riverine Zone |
|  | KE14 - Tana Riverine Zone |

ASAL: Arid and semi-arid lands

## **Combined zones map**


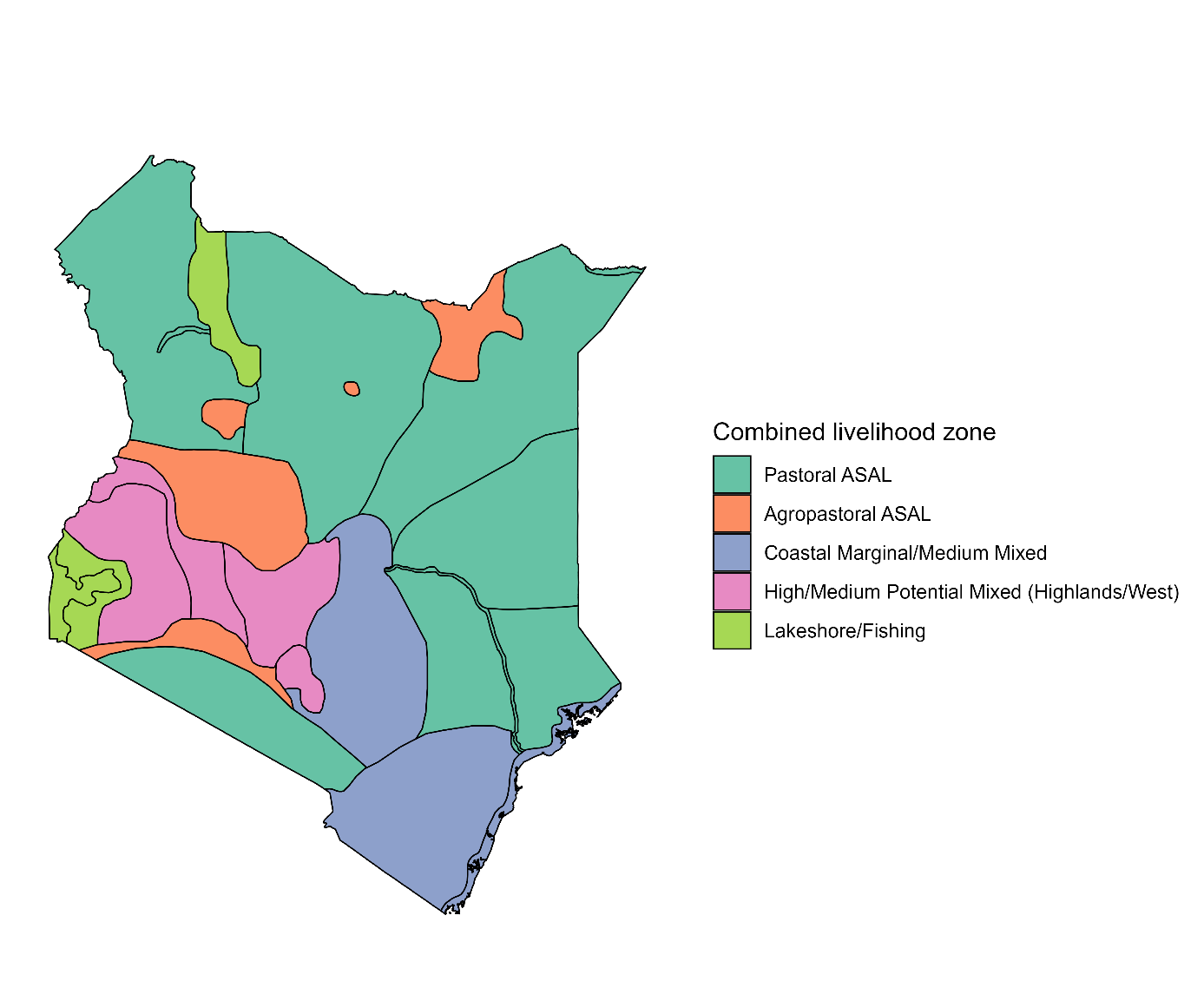


*Figure 17: The combined livelihood zones created for the network analyses.*

ASAL: Arid and semi-arid lands

# **Network analyses**

## **Edge weights**

*Table 2: Pooled seroprevalence by each livelihood zone-host cell. Estimates were used to weigh the edges in the bipartite network analysis. A default continuity correction of 0.5 was utilised to stabilise estimates from studies with a prevalence of zero.*

| **Combined livelihood zone** | **Host species** | **Records numbers** | **Total tested (n)** | **Pooled seroprevalence (95% CI)** | **τ²** | ***I*² (%)** |
| --- | --- | --- | --- | --- | --- | --- |
| Agropastoral ASAL | African buffalo | 1 | 10 | 4.5 (0.3-44.8) | 0 | 0 |
| Agropastoral ASAL | Camel | 5 | 1297 | 10.4 (2.3-36.0) | 1.28 | 95.1 |
| Agropastoral ASAL | Cattle | 11 | 3188 | 10.0 (6.3-15.5) | 0.47 | 91.47 |
| Agropastoral ASAL | Gazelle | 1 | 8 | 5.6 (0.3-50.5) | 0 | 0 |
| Agropastoral ASAL | Giraffe | 1 | 14 | 3.3 (0.2-36.6) | 0 | 0 |
| Agropastoral ASAL | Goat | 6 | 2608 | 12.5 (5.0-27.7) | 0.54 | 92.89 |
| Agropastoral ASAL | Human | 7 | 4234 | 15.3 (3.8-45.1) | 2.53 | 99.48 |
| Agropastoral ASAL | Impala | 1 | 36 | 1.4 (0.1-18.2) | 0 | 0 |
| Agropastoral ASAL | Sheep | 4 | 594 | 6.7 (2.4-17.6) | 0.24 | 60.54 |
| Coastal Marginal/Medium Mixed | Cattle | 2 | 175 | 8.7 (0.2-80.7) | 0.02 | 6.2 |
| Coastal Marginal/Medium Mixed | Goat | 2 | 161 | 2.3 (0.0-100.0) | 4.46 | 77.69 |
| Coastal Marginal/Medium Mixed | Human | 3 | 609 | 10.7 (4.7-22.7) | 0 | 0 |
| Coastal Marginal/Medium Mixed | Pig | 1 | 3 | 12.5 (0.7-73.4) | 0 | 0 |
| High/Medium Potential Mixed (Highlands/West) | Cattle | 14 | 8565 | 5.0 (2.7-9.2) | 0.92 | 96.63 |
| High/Medium Potential Mixed (Highlands/West) | Goat | 2 | 83 | 3.2 (0.0-100.0) | 3.86 | 63.66 |
| High/Medium Potential Mixed (Highlands/West) | Human | 14 | 6500 | 3.6 (1.5-8.1) | 2.04 | 95.54 |
| High/Medium Potential Mixed (Highlands/West) | Pig | 17 | 2045 | 1.2 (0.7-2.2) | 0.45 | 28.65 |
| High/Medium Potential Mixed (Highlands/West) | Sheep | 1 | 1 | 25.0 (1.3-89.1) | 0 | 0 |
| Lakeshore/Fishing | Pig | 1 | 23 | 2.1 (0.1-25.9) | 0 | 0 |
| Pastoral ASAL | African buffalo | 2 | 21 | 26.4 (0.2-98.7) | 0 | 0 |
| Pastoral ASAL | Camel | 4 | 491 | 16.0 (7.3-31.3) | 0.21 | 76.68 |
| Pastoral ASAL | Cattle | 16 | 12036 | 14.4 (10.5-19.4) | 0.42 | 96.84 |
| Pastoral ASAL | Eland | 1 | 14 | 14.3 (3.6-42.7) | 0 | 0 |
| Pastoral ASAL | Goat | 11 | 3340 | 4.7 (2.6-8.3) | 0.48 | 85.72 |
| Pastoral ASAL | Human | 13 | 6107 | 20.6 (10.9-35.6) | 1.40 | 99.01 |
| Pastoral ASAL | Oryx | 1 | 20 | 5.0 (0.7-28.2) | 0 | 0 |
| Pastoral ASAL | Pig | 1 | 6 | 7.1 (0.4-57.7) | 0 | 0 |
| Pastoral ASAL | Sheep | 8 | 2400 | 3.4 (1.6-6.8) | 0.54 | 80.93 |
| Pastoral ASAL | Wildebeest | 1 | 175 | 17.7 (12.7-24.1) | 0 | 0 |

ASAL: Arid and semi-arid lands, CI: Confidence intervals

## **Theoretical research gaps**

*Table 3: A summary of research gaps based on theoretically possible links that were missing from the bipartite network. Some combinations may therefore be unlikely in practice.*

| **Combined livelihood zones** | **Host** |
| --- | --- |
| Agropastoral ASAL | Eland |
| Agropastoral ASAL | Oryx |
| Agropastoral ASAL | Pig |
| Agropastoral ASAL | Wildebeest |
| Coastal Marginal/Medium Mixed | African buffalo |
| Coastal Marginal/Medium Mixed | Camel |
| Coastal Marginal/Medium Mixed | Eland |
| Coastal Marginal/Medium Mixed | Gazelle |
| Coastal Marginal/Medium Mixed | Giraffe |
| Coastal Marginal/Medium Mixed | Impala |
| Coastal Marginal/Medium Mixed | Oryx |
| Coastal Marginal/Medium Mixed | Sheep |
| Coastal Marginal/Medium Mixed | Wildebeest |
| High/Medium Potential Mixed (Highlands/West) | African buffalo |
| High/Medium Potential Mixed (Highlands/West) | Camel |
| High/Medium Potential Mixed (Highlands/West) | Eland |
| High/Medium Potential Mixed (Highlands/West) | Gazelle |
| High/Medium Potential Mixed (Highlands/West) | Giraffe |
| High/Medium Potential Mixed (Highlands/West) | Impala |
| High/Medium Potential Mixed (Highlands/West) | Oryx |
| High/Medium Potential Mixed (Highlands/West) | Wildebeest |
| Lakeshore/Fishing | African buffalo |
| Lakeshore/Fishing | Camel |
| Lakeshore/Fishing | Cattle |
| Lakeshore/Fishing | Eland |
| Lakeshore/Fishing | Gazelle |
| Lakeshore/Fishing | Giraffe |
| Lakeshore/Fishing | Goat |
| Lakeshore/Fishing | Human |
| Lakeshore/Fishing | Impala |
| Lakeshore/Fishing | Oryx |
| Lakeshore/Fishing | Sheep |
| Lakeshore/Fishing | Wildebeest |
| Pastoral ASAL | Gazelle |
| Pastoral ASAL | Giraffe |
| Pastoral ASAL | Impala |

ASAL: Arid and semi-arid lands

## **Study characteristics table**

Table 4: A summary of the species, samples and diagnostic tests investigated in the prevalence studies included in the systematic review

| **Host category** | **Species** | **Samples** | **Laboratory tests** | **References** |
| --- | --- | --- | --- | --- |
| **Domestic animals** | **Camels** | Blood | **CFT** | [1] |
|  |  |  | **ELISA** | [2-6] |
|  |  |  | **PCR** | [4, 7] |
|  |  |  | **RBPT; SAT; CFT** | [8] |
|  |  |  | **RBT** | [7] |
|  |  |  | **RBT; ELISA** | [9] |
|  |  |  | **RBT; SAT; CFT** | [10] |
|  |  | Milk | **MRT** | [11] |
|  |  |  | **PCR** | [12] |
|  |  | Pooled milk | **MRT** | [13, 14] |
|  |  |  | **PCR** | [12] |
|  | **Cattle** | Blood | **CFT** | [1, 11, 15] |
|  |  |  | **ELISA** | [2, 5, 6, 16-22] |
|  |  |  | **PCR** | [7] |
|  |  |  | **RBPT; SAT; CFT** | [23] |
|  |  |  | **RBT** | [7, 24-26] |
|  |  |  | **SAT; CFT** | [27] |
|  |  |  | **RBT; ELISA** | [9, 13, 28] |
|  |  |  | **RBT; SAT; CFT** | [10] |
|  |  |  | **Rapid assay** | [29] |
|  |  | Milk | **ELISA** | [12] |
|  |  |  | **MRT** | [11, 24, 30] |
|  |  |  | **PCR** | [12] |
|  |  | Pooled milk | **ELISA** | [12] |
|  |  |  | **MRT** | [13, 30-33] |
|  |  |  | **PCR** | [12] |
|  |  |  | **MRT; ELISA** | [34, 35] |
|  | **Donkey** | Blood | **PCR** | [36] |
|  | **Goat** | Blood | **CFT** | [11] |
|  |  |  | **ELISA** | [2, 3, 5, 6, 18, 19, 28] |
|  |  |  | **PCR** | [7, 36] |
|  |  |  | **RBPT; SAT; CFT** | [23] |
|  |  |  | **RBT** | [7, 26] |
|  |  |  | **SAT** | [37] |
|  |  |  | **RBT; ELISA** | [9] |
|  |  |  | **RBT; ELISA; CFT** | [38] |
|  |  |  | **RBT; SAT; CFT** | [10] |
|  |  | Cervical swab | **Culture** | [37] |
|  |  | Milk | **Culture** | [37] |
|  |  |  | **MRT** | [11] |
|  |  |  | **PCR** | [12] |
|  |  | Pooled milk | **MRT** | [13] |
|  |  |  | **PCR** | [12] |
|  | **Pig** | Blood | **CFT; SAT; RBT** | [39] |
|  |  |  | **PCR** | [40] |
|  |  |  | **RBT** | [40] |
|  | **Sheep** | Blood | **CFT** | [11] |
|  |  |  | **ELISA** | [2, 3, 5, 6, 18, 19, 28] |
|  |  |  | **PCR** | [7, 36] |
|  |  |  | **RBPT; SAT; CFT** | [23] |
|  |  |  | **RBT** | [7, 26] |
|  |  |  | **RBT; ELISA** | [9] |
|  |  |  | **RBT; ELISA; CFT** | [38] |
|  |  |  | **RBT; SAT; CFT** | [10] |
|  |  | Milk | **MRT** | [11, 12] |
|  |  | Pooled milk | **PCR** | [12] |
|  |  | Semen | **Culture; CFT** | [41] |
| **Humans** | **Human (community, occupationally exposed, or HIV-positive)** | Blood | **Agglutination test** | [42] |
|  |  |  | **ELISA** | [2, 3, 5, 6, 18, 43-45] |
|  |  |  | **PCR** | [2, 7, 13] |
|  |  |  | **RBT** | [7] |
|  |  |  | **SAT; TAT** | [46] |
|  |  |  | **Rapid assay** | [29] |
|  |  | Serum | **ELISA** | [47] |
|  |  |  | **RBPT; SAT; CFT** | [48] |
|  |  |  | **RBT; ELISA** | [11] |
|  | **Human (febrile patients)** | Blood | **ELISA** | [19, 49, 50] |
|  |  |  | **FBAT** | [13, 51] |
|  |  |  | **LFT** | [52] |
|  |  |  | **PCR** | [49, 53-57] |
|  |  |  | **RBT** | [56-61] |
|  |  |  | **PCR; RBT; ELISA** | [62] |
| **Vectors** | **Hippoboscid flies** | Tissue homogenate | **PCR** | [36] |
| **Wildlife** | **African buffalo** | Blood | **SAT; CFT** | [63] |
|  |  |  | **RBT; SAT; CFT** | [10] |
|  | **Blue wildebeest** | Blood | **SAT; CFT** | [63] |
|  | **Buffalo** | Blood | **CFT** | [1] |
|  |  |  | **ELISA** | [64] |
|  |  |  | **PCR** | [64] |
|  | **Cheetah** | Blood | **ELISA** | [64] |
|  |  |  | **PCR** | [64] |
|  | **Eland** | Blood | **ELISA** | [64] |
|  |  |  | **PCR** | [64] |
|  |  |  | **RBT; SAT; CFT** | [10] |
|  | **Elephant** | Blood | **ELISA** | [64] |
|  |  |  | **PCR** | [64] |
|  | **Gazelle** | Blood | **CFT** | [1] |
|  |  |  | **ELISA** | [64] |
|  | **Giraffe** | Blood | **CFT** | [1] |
|  |  |  | **ELISA** | [64] |
|  |  |  | **PCR** | [64] |
|  | **Hartebeest** | Blood | **ELISA** | [64] |
|  | **Impala** | Blood | **CFT** | [1] |
|  |  |  | **ELISA** | [64] |
|  | **Leopard** | Blood | **ELISA** | [64] |
|  | **Lion** | Blood | **ELISA** | [64] |
|  |  |  | **PCR** | [64] |
|  | **Oryx** | Blood | **ELISA** | [64] |
|  |  |  | **PCR** | [64] |
|  |  |  | **RBT; SAT; CFT** | [10] |
|  | **Rhino** | Blood | **ELISA** | [64] |
|  | **Rhinoceros** | Blood | **CFT** | [65] |
|  | **Warthog** | Blood | **ELISA** | [64] |
|  |  |  | **PCR** | [64] |
|  | **Waterbuck** | Blood | **ELISA** | [64] |
|  | **Wildebeest** | Blood | **ELISA** | [64] |
|  | **Zebra** | Blood | **ELISA** | [64] |

BMAT, SAT, TAT: Micro-, Serum and Tube agglutination tests, CFT: Complement fixation test, ELISA: Enzyme-linked immunosorbent assay, LFT: Lateral flow test, FBAT: Febrile *Brucella* antigen test, MRT: Milk ring test, PCR: Polymerase chain reaction, Rapid test: Rapid immunochromatographic flow assay, RBT: Rose Bengal test

## **Bibliography**

1. Kimber KR, Lubroth J, Dubovi EJ, Berninger ML, Demaar TW. Serologic survey of selected viral, bacterial, and protozoal agents in captive and free-ranging ungulates from central Kenya. Annals of the New York Academy of Sciences. 2002;969(1):217-23. doi: 10.1111/j.1749-6632.2002.tb04382.x.

2. Lokamar PN, Kutwah MA, Munde EO, Oloo D, Atieli H, Gumo S, et al. Prevalence of brucellosis in livestock keepers and domestic ruminants in Baringo County, Kenya. PLOS Glob Public Health. 2022;2(8). doi: 10.1371/journal.pgph.0000682. PubMed PMID: rayyan-295363253.

3. Muema J, Oboge H, Mutono N, Makori A, Oyugi J, Bukania Z, et al. Sero - epidemiology of brucellosis in people and their livestock: A linked human - animal cross-sectional study in a pastoralist community in Kenya. Front Vet Sci. 2022;9:1031639. doi: 10.3389/fvets.2022.1031639. PubMed PMID: rayyan-295362793.

4. Muturi M, Akoko J, Nthiwa D, Chege B, Nyamota R, Mutiiria M, et al. Serological evidence of single and mixed infections of Rift Valley fever virus, Brucella spp. and Coxiella burnetii in dromedary camels in Kenya. PLoS neglected tropical diseases. 2021;15(3):e0009275. doi: 10.1371/journal.pntd.0009275. PubMed PMID: rayyan-295362798.

5. Mwatondo A, Muturi M, Akoko J, Nyamota R, Nthiwa D, Maina J, et al. Seroprevalence and related risk factors of *Brucella* spp. in livestock and humans in Garbatula subcounty, Isiolo county, Kenya. PLoS neglected tropical diseases. 2023;17(10):e0011682. doi: 10.1371/journal.pntd.0011682. PubMed PMID: rayyan-295362743.

6. Osoro EM, Munyua P, Omulo S, Ogola E, Ade F, Mbatha P, et al. Strong Association Between Human and Animal *Brucella* Seropositivity in a Linked Study in Kenya, 2012-2013. Am J Trop Med Hyg. 2015;93(2):224-31. doi: 10.4269/ajtmh.15-0113. PubMed PMID: rayyan-295362733.

7. Akoko JM, Pelle R, Lukambagire AS, Machuka EM, Nthiwa D, Mathew C, et al. Molecular epidemiology of *Brucella* species in mixed livestock-human ecosystems in Kenya. Sci Rep. 2021;11(1):8881. doi: 10.1038/s41598-021-88327-z. PubMed PMID: rayyan-295362713.

8. Waghela S, Fazil MA, Gathuma JM, Kagunya DK. A serological survey of brucellosis in camels in north-eastern province of Kenya. Tropical Animal Health and Production. 1978;10(1):28-9. doi: 10.1007/BF02235298. PubMed PMID: rayyan-295365114.

9. Njeru J, Nthiwa D, Akoko J, Oyas H, Bett B. Incidence of *Brucella* infection in various livestock species raised under the pastoral production system in Isiolo County, Kenya. BMC Vet Res. 2021;17(1):342. doi: 10.1186/s12917-021-03036-z. PubMed PMID: rayyan-295362737.

10. Paling RW, Waghela S, Macowan KJ, Heath BR. The occurrence of infectious diseases in mixed farming of domesticated wild herbivores and livestock in Kenya. II. Bacterial diseases. J Wildl Dis. 1988;24(2):308-16. doi: 10.7589/0090-3558-24.2.308. PubMed PMID: rayyan-295362880.

11. Chota AC, Magwisha HB, Stella B, Bunuma EK, Shirima GM, Mugambi JM, et al. Prevalence of brucellosis in livestock and incidences in humans in east Africa. African Crop Science Journal. 2016;24(1):45-52. doi: 10.4314/acsj.v24i1.5S.

12. Wainaina M, Aboge GO, Omwenga I, Ngaywa C, Ngwili N, Kiara H, et al. Detection of *Brucella* spp. in raw milk from various livestock species raised under pastoral production systems in Isiolo and Marsabit Counties, northern Kenya. TROPICAL ANIMAL HEALTH AND PRODUCTION. 2020;52(6):3537-44. doi: 10.1007/s11250-020-02389-1. PubMed PMID: rayyan-295362044.

13. Karlsson PA, Persson C, Akoko J, Bett B, Lundkvist Å, Lindahl JF. Using a One Health Case-Based Investigation for Improved Control of Brucellosis in Isiolo, Kenya. Front Trop Dis. 2021;2. doi: 10.3389/fitd.2021.711425. PubMed PMID: rayyan-295363658.

14. Noor M, Rotich V, Kiarie JW, Cheruiyot K, Kagira JM. Prevalence, risk factors associated with brucellosis and presence of pathogenic bacteria isolated from camel milk in Garissa County, Kenya. South Asian Journal of Research in Microbiology. 2020;6(4):42-52. doi: 10.9734/SAJRM/2020/v6i430158.

15. Gössler R, Leyk W, Hünermund G. [Serological studies in cattle in the Kabete area (Kenya). 1. Occurrence of antibodies against para influenza-3-,IBR-,BDD-virus, chlamydia and Coxiella burneti]. Berl Munch Tierarztl Wochenschr. 1973;86(9):164-6. Epub 1973/05/01. PubMed Central PMCID: PMC4352863.

16. Akoko JM, Mwatondo A, Muturi M, Wambua L, Abkallo HM, Nyamota R, et al. Mapping brucellosis risk in Kenya and its implications for control strategies in sub-Saharan Africa. Sci Rep. 2023;13(1):20192. doi: 10.1038/s41598-023-47628-1. PubMed PMID: rayyan-295362699.

17. Kadohira M, McDermott JJ, Shoukri MM, Thorburn MA. Assessing infections at multiple levels of aggregation. Prev Vet Med. 1997;29(3):161-77. doi: 10.1016/S0167-5877(96)01084-7. PubMed PMID: rayyan-295364992.

18. Kairu-Wanyoike S, Nyamwaya D, Wainaina M, Lindahl J, Ontiri E, Bukachi S, et al. Positive association between *Brucella* spp. seroprevalences in livestock and humans from a cross-sectional study in Garissa and Tana River Counties, Kenya. PLoS neglected tropical diseases. 2019;13(10):e0007506. doi: 10.1371/journal.pntd.0007506. PubMed PMID: rayyan-295362871.

19. Nakeel MJ, Arimi SM, Kitala P, Nduhiu G, Njenga JM, Wabacha JK. A sero-epidemiological survey of brucellosis, Q-fever and leptospirosis in livestock and humans and associated risk factors in kajiado county-Kenya. J Trop Dis. 2016;4(3):8. doi: 10.4172/2329-891X.1000215.

20. Nthiwa D, Alonso S, Odongo D, Kenya E, Bett B. Zoonotic Pathogen Seroprevalence in Cattle in a Wildlife-Livestock Interface, Kenya. Ecohealth. 2019;16(4):712-25. doi: 10.1007/s10393-019-01453-z. PubMed PMID: rayyan-295362724.

21. Okumu TA, John NM, Wabacha JK, Tsuma V, VanLeeuwen J. Seroprevalence of antibodies for bovine viral diarrhoea virus, *Brucella abortus* and *Neospora caninum*, and their roles in the incidence of abortion/foetal loss in dairy cattle herds in Nakuru District, Kenya. BMC Vet Res. 2019;15(1):95. doi: 10.1186/s12917-019-1842-8. PubMed PMID: rayyan-295362890.

22. Rajeev M, Mutinda M, Ezenwa VO. Pathogen Exposure in Cattle at the Livestock-Wildlife Interface. Ecohealth. 2017;14(3):542-51. doi: 10.1007/s10393-017-1242-0. PubMed PMID: rayyan-295362739.

23. Ndarathi CM, Waghela S. Brucellosis in Maasai livestock in Kajiado district in Kenya. Indian Journal of Animal Sciences. 1991;61(2):156–7.

24. Gicheru MN, Mwangi E. Prevalence and Knowledge of Brucellosis in Dairy Cattle in Makuyu Division, Murang'a County, Kenya. International Journal of Scientific Engineering and Technology. 2015;4(12):549-55. doi: 10.17950/ijset/v4s12/1202.

25. Kagumba M, Nandokha E. A survey of the prevalence of bovine brucellosis in East Africa. Bulletin of Animal Health and Production in Africa. 1978;26(3):224–9.

26. Kagunya DKJ, Waiyaki PG. A serological survey of animal brucellosis in the north-eastern province of Kenya. Kenya Veterinarian. 1978;2(2):35–8.

27. Nagy LK, Sorheim AO. A survey of *Brucella* infection of cattle in Kenya. A comparison of four serological tests for the diagnosis of infection. Vet Rec. 3 ed. England1969. p. 65-7.

28. Odongo MO, Bebora LC, Gathumbi JK, Aboge GO, Waiboci LW, Erume J. Seroprevalence and spatial distribution of livestock brucellosis using three serological tests in Kajiado County, Kenya. OPEN VETERINARY JOURNAL. 2023;13(12):1583-96. doi: 10.5455/OVJ.2023.v13.i12.8 WE - Emerging Sources Citation Index (ESCI). PubMed PMID: rayyan-295362020.

29. Fèvre EM, de Glanville WA, Thomas LF, Cook EAJ, Kariuki S, Wamae CN. An integrated study of human and animal infectious disease in the Lake Victoria crescent small-holder crop-livestock production system, Kenya. BMC Infect Dis. 2017;17(1):457. doi: 10.1186/s12879-017-2559-6. PubMed PMID: rayyan-295362866.

30. Njuguna JN, Gicheru MM, Kamau LM, Mbatha PM. Incidence and knowledge of bovine brucellosis in Kahuro district, Murang'a County, Kenya. Tropical animal health and production. 2017;49(5):1035-40. doi: 10.1007/s11250-017-1296-6. PubMed PMID: rayyan-295362757.

31. Kagira JM, Hussein A, Kiptanui A, Lkurasian L, Kiarie J, Cheruiyot K. Risk factors associated with prevalence of brucellosis and bacteria in fermented cow milk obtained from Kajiado Central Sub-County in Kenya. Asian Journal of Research in Animal and Veterinary Sciences. 2023;6(4):441-8. doi: 10.9734/ajravs/2023/v6i4272.

32. Kang'ethe EK, Arimi SM, Omore AO, McDermott JJ, Nduhiu JG, Macharia JK, et al. Testing for antibodies to Brucella abortus in milk from consumers and market agents in Kenya using milk ring test and enzyme immunoassay. Kenya Veterinarian. 2004;27:18-21. PubMed PMID: rayyan-295394227.

33. Namanda AT, Kakai R, Otsyula M. The role of unpasteurized "hawked" milk in the transmission of brucellosis in Eldoret municipality, Kenya. J Infect Dev Ctries. 2009;3(4):260-6. doi: 10.3855/jidc.122. PubMed PMID: rayyan-295364721.

34. Arimi SM, Koroti E, Kang'ethe EK, Omore AO, McDermott JJ. Risk of infection with B*rucella abortus* and *Escherichia coli* O157:H7 associated with marketing of unpasteurized milk in Kenya. Acta Trop. 2005;96(1):1-8. doi: 10.1016/j.actatropica.2005.05.012. PubMed PMID: rayyan-295362905.

35. Kang'ethe EK, Ekuttan CE, Kimani VN, Kiragu MW. Investigations into the prevalence of bovine brucellosis and the risk factors that predispose humans to infection among urban dairy and non-dairy farming households in Dagoretti Division, Nairobi, Kenya. East African medical journal. 2007;84(11):S96-100. doi: 10.4314/eamj.v84i11.9583. PubMed PMID: rayyan-295362897.

36. Mwaki DM, Kidambasi KO, Kinyua J, Ogila K, Kigen C, Getange D, et al. Molecular detection of novel *Anaplasma* sp . and zoonotic hemopathogens in livestock and their hematophagous biting keds (genus *Hippobosca*) from Laisamis, northern Kenya. Open Res Afr. 2022;5:23. doi: 10.12688/openresafrica.13404.1. PubMed PMID: rayyan-295362790.

37. Philpott M, Auko O. Caprine brucellosis in Kenya. Br Vet J. 1972;128(12):642-51. doi: 10.1016/s0007-1935(17)36637-x. PubMed PMID: rayyan-295362933.

38. Wainaina M, Lindahl JF, Dohoo I, Mayer-Scholl A, Roesel K, Mbotha D, et al. Longitudinal Study of Selected Bacterial Zoonoses in Small Ruminants in Tana River County, Kenya. Microorganisms. 2022;10(8). doi: 10.3390/microorganisms10081546. PubMed PMID: rayyan-295362852.

39. Waghela S, Gathuma JM. A serological survey of the prevalence of brucellosis in pigs in Kenya. Bulletin of animal health and production in Africa Bulletin des santé et production animales en Afrique. 1976;24(3):251-3. PubMed PMID: rayyan-295365098.

40. Akoko J, Pelle R, Kivali V, Schelling E, Shirima G, Machuka EM, et al. Serological and molecular evidence of *Brucella* species in the rapidly growing pig sector in Kenya. BMC Vet Res. 2020;16(1):133. doi: 10.1186/s12917-020-02346-y. PubMed PMID: rayyan-295362784.

41. Cameron RD, Carles AB, Lauerman LH, Jr. The incidence of Brucella ovis in some Kenya flocks and its relationship to clinical lesions and semen quality. Vet Rec. 1971;89(21):552-7. doi: 10.1136/vr.89.21.552. PubMed PMID: rayyan-295363049.

42. Jumba MM, Mirza NB, Mwaura FB. Agglutinins for brucellae antigens in blood sera of an urban and rural population in Kenya. East African medical journal. 1996;73:204-6. Epub 3. PubMed PMID: rayyan-295363057.

43. Kahariri S, Kitala PM, Muchemi GM, Njenga K, Nanyingi M. Sero-prevalence and risk factors for human brucellosis in Marsabit County, Kenya (2014). Pan Afr Med J One Health. 2021;4. doi: 10.11604/pamj-oh.2021.4.9.27024. PubMed PMID: rayyan-295363624.

44. Nyamota R, Maina J, Akoko J, Nthiwa D, Mwatondo A, Muturi M, et al. Seroprevalence of *Brucella* spp. and Rift Valley fever virus among slaughterhouse workers in Isiolo County, northern Kenya. PLoS neglected tropical diseases. 2023;17(10):e0011677. doi: 10.1371/journal.pntd.0011677. PubMed PMID: rayyan-295362741.

45. Omballa VO, Musyoka RN, Vittor AY, Wamburu KB, Wachira CM, Waiboci LW, et al. Serologic evidence of the geographic distribution of bacterial zoonotic agents in Kenya, 2007. Am J Trop Med Hyg. 2016;94(1):43-51. doi: 10.4269/ajtmh.15-0320. PubMed PMID: rayyan-295364333.

46. Ndinya-Achola JO, Nsanzumuhire H, Okelo GBA. Some possible infectious hazards due to blood transfusion in Nairobi. East African Medical Journal. 1980;57(1):55-9. PubMed PMID: rayyan-295365105.

47. Paul J, Gilks C, Batchelor B, Ojoo J, Amir M, Selkon JB. Serological responses to brucellosis in HIV-seropositive patients. Trans R Soc Trop Med Hyg. 1995;89(2):228-30. doi: 10.1016/0035-9203(95)90508-1.

48. Oomen LJ, Waghela S. The rose bengal plate test in human brucellosis. Trop Geogr Med. 1974;26(3):300-2. Epub 1974/09/01. PubMed Central PMCID: PMC4439467.

49. Munyua P, Osoro E, Hunsperger E, Ngere I, Muturi M, Mwatondo A, et al. High incidence of human brucellosis in a rural pastoralist community in Kenya, 2015. PLoS Neglected Tropical Diseases. 2021;15(2). doi: 10.1371/journal.pntd.0009049. PubMed PMID: rayyan-295363642.

50. Nyataya J, Maraka M, Lemtudo A, Masakhwe C, Mutai B, Njaanake K, et al. Serological Evidence of Yersiniosis, Tick-Borne Encephalitis, West Nile, Hepatitis E, Crimean-Congo Hemorrhagic Fever, Lyme Borreliosis, and Brucellosis in Febrile Patients Presenting at Diverse Hospitals in Kenya. Vector Borne Zoonotic Dis. 2020;20(5):348-57. doi: 10.1089/vbz.2019.2484. PubMed PMID: rayyan-295362870.

51. Maiyo G. Distribution and prevalence of human brucellosis among patients reporting at Chemundu Dispensary, Nandi County, Kenya. Baraton Interdisciplinary Research Journal. 2016;6:73-82.

52. Smith S, Koech R, Nzorubara D, Otieno M, Wong L, Bhat G, et al. Connected diagnostics: linking digital rapid diagnostic tests and mobile health wallets to diagnose and treat brucellosis in Samburu, Kenya. BMC Med Inform Decis Mak. 2019;19(1):139. doi: 10.1186/s12911-019-0854-4. PubMed PMID: rayyan-295362872.

53. Kiambi SG, Fèvre EM, Omolo J, Oundo J, de Glanville WA. Risk factors for acute human brucellosis in Ijara, north-eastern Kenya. PLoS neglected tropical diseases. 2020;14(4):e0008108. doi: 10.1371/journal.pntd.0008108. PubMed PMID: rayyan-295362719.

54. Njoroge RN, Wurapa EK, Waitumbi JN, Breiman RF, Kariuki Njenga M. The etiology of acute febrile illness in patients presenting to Garissa Provincial Hospital in Northeastern Province, Kenya. Am J Trop Med Hyg. 2011;85(6):136. PubMed PMID: rayyan-295362853.

55. Verani JR, eno EN, Hunsperger EA, Munyua P, Osoro E, Marwanga D, et al. Acute febrile illness in Kenya: Clinical characteristics and pathogens detected among patients hospitalized with fever, 2017–2019. PLoS ONE. 2024;19(8):e0305700. doi: 10.1371/journal.pone.0305700.

56. Wainaina M, Lindahl JF, Mayer-Scholl A, Ufermann CM, Domelevo Entfellner JB, Roesler U, et al. Molecular and serological diagnosis of multiple bacterial zoonoses in febrile outpatients in Garissa County, north-eastern Kenya. Sci Rep. 2024;14(1):12263. doi: 10.1038/s41598-024-62714-8. PubMed PMID: rayyan-295362868.

57. Waringa NMA, Waiboci LW, Bebora L, Kinyanjui PW, Kosgei P, Kiambi S, et al. Human brucellosis in Baringo County, Kenya: Evaluating the diagnostic kits used and identifying infecting Brucella species. PLoS ONE. 2023;18(1):e0269831. doi: 10.1371/journal.pone.0269831. PubMed PMID: rayyan-295362731.

58. Alumasa L, Thomasid LF, Amanya F, Njorogeid SM, Moriyónid I, Makhandiaid J, et al. Hospital-based evidence on cost-effectiveness of brucellosis diagnostic tests and treatment in kenyan hospitals. PLoS Neglected Tropical Diseases. 2021;15(1):1-19. doi: 10.1371/journal.pntd.0008977. PubMed PMID: rayyan-295363633.

59. de Glanville WA, Conde-Álvarez R, Moriyón I, Njeru J, Díaz R, Cook EAJ, et al. Poor performance of the rapid test for human brucellosis in health facilities in Kenya. PLoS neglected tropical diseases. 2017;11(4):e0005508. doi: 10.1371/journal.pntd.0005508. PubMed PMID: rayyan-295362833.

60. Maichomo MW, McDermott JJ, Arimi SM, Gathura PB. Assessment of the Rose-Bengal plate test for the diagnosis of human brucellosis in health facilities in Narok district, Kenya. East African Medical Journal. 1998;75(4):219-22. PubMed PMID: rayyan-295365049.

61. Muriuki SMK, McDermott JJ, Arimi SM, Mugambi JTM, Wamola IA. Criteria for better detection of brucellosis in the Narok district of Kenya. East African Medical Journal. 1997;74(5):317-20. PubMed PMID: rayyan-295365081.

62. Njeru J, Melzer F, Wareth G, El-Adawy H, Henning K, Pletz MW, et al. Human Brucellosis in Febrile Patients Seeking Treatment at Remote Hospitals, Northeastern Kenya, 2014-2015. Emerging infectious diseases. United States2016. p. 2160-4.

63. Waghela S, Karstad L. Antibodies to *Brucella* spp. among blue wildebeest and African buffalo in Kenya. Journal of wildlife diseases. 1986;22(2):189-92. doi: 10.7589/0090-3558-22.2.189. PubMed PMID: rayyan-295365117.

64. Gakuya F, Akoko J, Wambua L, Nyamota R, Ronoh B, Lekolool I, et al. Evidence of co-exposure with *Brucella* spp, *Coxiella burnetii*, and Rift Valley fever virus among various species of wildlife in Kenya. PLoS neglected tropical diseases. 2022;16(8):e0010596. doi: 10.1371/journal.pntd.0010596. PubMed PMID: rayyan-295362788.

65. Fischer-Tenhagen C, Hamblin C, Quandt S, Frölich K. Serosurvey for selected infectious disease agents in free-ranging black and white rhinoceros in Africa. Journal of wildlife diseases. 2000;36(2):316-23. doi: 10.7589/0090-3558-36.2.316. PubMed PMID: rayyan-295362935.
